# Manipulating Interactions between Dielectric Particles with Electric Fields: A General Electrostatic Many-Body Framework

**DOI:** 10.1021/acs.jctc.2c00008

**Published:** 2022-09-08

**Authors:** Muhammad Hassan, Connor Williamson, Joshua Baptiste, Stefanie Braun, Anthony J. Stace, Elena Besley, Benjamin Stamm

**Affiliations:** † Sorbonne Université, CNRS, Université de Paris, Laboratoire Jacques-Louis Lions (LJLL), F-75005Paris, France; ‡ School of Chemistry, University of Nottingham, University Park, NG7 2RD, United Kingdom; § Institute of Applied Analysis and Numerical Simulation, University of Stuttgart, Pfaffenwaldring 57, 70569Stuttgart, Germany

## Abstract

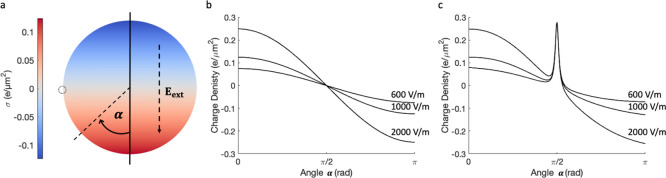

We derive a rigorous analytical formalism and propose a numerical method for the quantitative evaluation of the electrostatic interactions between dielectric particles in an external electric field. This formalism also allows for inhomogeneous charge distributions, and, in particular, for the presence of pointlike charges on the particle surface. The theory is based on a boundary integral equation framework and yields analytical expressions for the interaction energy and net forces that can be computed in linear scaling cost, with respect to the number of interacting particles. We include numerical results that validate the proposed method and show the limitations of the fixed dipole approximation at small separation between interacting particles. The proposed method is also applied to study the stability and melting of ionic colloidal crystals in an external electric field.

## 1 Introduction

The ability to control the behavior and interactions of neutral and charged particles with externally applied electric fields has significant implications for many areas of fundamental and applied science.^1−10^ The use of electrospray to promote the surface assembly of nanoparticle films has been shown to yield regular arrays when undertaken in the presence of an electric field.^1^ The application of an electric field during the preparation of organic solar cells by spray deposition has been found to improve the efficiency of power conversion.^2^ In electrostatic powder coating, the presence of an electric field can improve particle transfer efficiency and the control of film thickness.^3^ Electric fields can also facilitate the separation and removal of charged particles from such environments as the flue gas in coal-fired power stations.^4^ The self-assembly, interactions, structure, and dynamics of colloidal suspensions^11^ and binary nanoparticle crystals^12^ can be manipulated in a controlled—and often reversible—manner, using external electric fields.

Upon exposure to an external electric field, a dipole moment is induced on particles that may lead to a dramatic change in the macroscopic properties of their assemblies or suspensions. In suspensions, an applied external electric field may cause electrorheological effects where the viscosity of a suspension increases by several orders of magnitude leading to a liquid–solid phase transition, which is typically reversed as soon as the field is removed.^13,14^ Dipole interactions induced by an applied field will alter the structure of a suspension causing changes in flow behavior. The possibility of rapid switching from one state to another has led to a variety of industrial applications, including nanoparticle-based displays^5^ and the use of Janus particles in biomedical applications and computer screens.^6^ Janus particles, as both solid and liquid droplets, have demonstrated their potential for microsensors and actuators, microfluidics applications, and the stabilization of emulsions.^7^ It has been demonstrated that Janus particles can be activated, oriented,^8,9^ manipulated,^10^ and rotated^7^ by an electric field.

An advantage of manipulating particle interactions by an applied electric field is that it does not require additional chemical modifications of the solvent or the particles, and the interactions remain adjustable, fully reversible, and instantaneous. The interaction of charged dielectric (polarizable) particles with an external electric field represents an additional contribution to the electrostatic interaction energy, which consists of Coulomb terms and charge-induced, many-body, multipolar interactions. In contrast to isotropic Coulomb forces, induced many-body multipolar interactions are anisotropic in nature and can give rise to unique crystalline and noncrystalline structures, especially in an applied electric field. These induced charge interactions play an important role in a variety of fundamental processes, such as the nucleation, growth, and melting of crystals, glass transitions, and various interfacial phenomena.^15−19^ Chemical and biological examples include atmospheric processes, such as dust particle agglomeration^20^ and aerosol growth^21^ in the planetary environments, the accumulation of red blood cells,^22^ and the assembly of colloidal particles in dilute solutions.^23^ In many of these applications, charge may accumulate at certain positions on the surfaces of particles (functional groups, structure defects, etc.), which can be represented by surface point charges.

Analytical methods for the accurate prediction of electrostatic interactions between dielectric particles are mainly restricted to the case of two particles. In the special cases of axial symmetry, an exact analytical solution of the two-body problem can be derived.^24,25^ Analytical solutions have also been derived for simple two-body problems involving surface point charges in vacuum^26^ and in the presence of external solvents.^27−30^ However, the two-body expansion series of the electrostatic potential must be truncated, which ultimately yields an approximation. By analogy with the mean-field theory, local expansions of the many-body problem (see, e.g., ref (31)) have also been suggested. These expansions reduce the problem to a one-body system by considering the effect of the electric field induced by all but one particle, and by solving the one-body electrostatic problem for each particle iteratively and self-consistently until the desired convergence is achieved. While this approach yields some insight into the description of a many-body system at a low computational cost, the iterative procedure can fail to converge especially at short separation. A mathematically more rigorous approach is to start with a global many-body formulation of the problem and interpret the many-body expansions as a block-Jacobi iteration scheme, where each block corresponds to one particle.

In a many-body formalism, the interaction of several dielectric particles can be described by a generalized Poisson equation, which, in turn, can be reduced to a boundary integral equation (BIE) representing the induced surface charge on the particles. Numerical methods such as the Boundary Element Method (BEM)^32,33^ or the Method of Moments (MoM)^34−36^ can be viewed as a discretization of an appropriate BIE. The method of image charges^37−40^ can be also used or combined with MoM to offer a hybrid discretization approach.^41^ Nevertheless, it is important to provide a rigorous characterization and mathematical framework of the exact solution, which contains no discretization errors. A mathematically well-founded approach to this problem has been proposed by Lindgren et al.,^42^ which formulates the many-body electrostatic problem, in terms of a BIE of the second type and uses a spectral Galerkin approximation to solve the resulting equations. This mathematical formalism allows for a rigorous convergence and complexity analysis of the induced surface charge, electrostatic interaction energy, and net forces acting on each particle (see refs (43−45)), since the continuous solution and the Galerkin approximation are both well characterized. In the same contribution, it is mathematically proven that (i) the proposed method scales linearly in computational cost, with respect to the number of particles, and (ii) the approximation error does not degrade as the system size increases.

This paper extends the framework of Lindgren et al.^42^ to include two fundamentally different physical effects, namely, the interaction of a many-body system with an external electric field and the presence of localized charge on the surface of a particle as described by point charges. The inclusion of these effects adds significant complexity to the mathematical model due to the nondecaying character of the external electric potential that does not vanish at infinity and the presence of singularities arising in the context of surface point charges. However, incorporating these important effects into the existing methodology broadens considerably its applicability and provides a versatile method for studying many important physical, chemical, and industrial processes previously inaccessible to accurate computation. Part of this work was conducted within the thesis by Baptiste.^57^

An additional aspect of this work is a derivation of the electrostatic interaction energy that is based only on quantities defined on the surfaces of particles, such that the negative gradient of this expression, with respect to the positions of particle, yields the electrostatic force. The developed formalism can explain mechanisms underpinning the structural stabilization of ionic colloidal crystals and their melting in an external electric field. Colloidal suspensions are widely used to study phase behavior in real space as the constituent nanometer- to micrometer-sized particles can be observed directly.^16,46−48^ Versatile model colloidal systems of charged polymethyl methacrylate (PMMA) particles have been studied comprehensively in the literature, because of the large range of size, charge, and structure that can be formed,^49−51^ and their structures are analogous to atomic and molecular crystals, with regard to symmetry and phase behavior.

Leunissen et al.^52^ showed that electrostatic interactions between PMMA particles of opposite charges can be tuned to form a diverse range of unique binary crystal structures. They demonstrated that these soft colloidal structures can be manipulated in a controlled and often reversible way using an external electric field, much as previously reported for electronic ink.^53^ The model proposed here is capable of quantitative predictions of many-body electrostatic interactions in an applied external electric field and reveals the fundamental principles driving the formation of interesting patterns in PMMA colloidal suspensions, as observed by Leunissen et al.^52^

The presented work is organized as follows. In Section 2, we describe the basic concepts of the many-body electrostatic problem and introduce the methodology (Subsections 2.1–2.3), which we extend to derive a single general expression for the electrostatic interaction energy between particles containing localized surface point charges and in the presence of an external electric field (Subsection 2.4). In calculations, these two additional features can be used independently. In Section 3, we present numerical results validating our method and show the limitations of the induced fixed dipoles approximation (Subsection 3.1). The proposed method is then applied to study the stability and melting of ionic colloidal crystals in an external electric field (Subsection 3.2). Final remarks and conclusions are followed by two appendices containing additional mathematical considerations and proofs.

## 2 Formulation of the Electrostatic Many-Body Framework

We consider a physical system of *N* nonoverlapping dielectric spherical particles, defined by their radii , centers , and dielectric constants , immersed in a background medium (solvent) that has a dielectric constant of κ_0_ > 0. The many-body system is considered at rest. The spherical particles are described as open balls denoted by  with surfaces . The surfaces of the dielectric particles represent the boundary ∂Ω between the interior Ω^–^ and the exterior Ω^+^ of the particles. We assume that (i) this surface ∂Ω carries a given free charge distribution σ_*f*_ and (ii) there is no charge in the interior of the particles, i.e., in Ω^–^ (See Section 1 of the Supporting Information for a precise mathematical description of these quantities). To account for the point-charge contribution to the surface free charge, the free charge σ_f_ is split into two contributions:
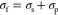
1Here, σ_s_ ∈ *L*^2^(Ω) corresponds to the square-integrable part of the surface charge, whereas σ_p_ is the point-charge contribution to the free charge represented by a linear combination of one or several Dirac delta distributions per particle:
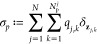
2where
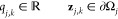
for all *j* = 1,..., *N* and *k* = 1, ..., *N_p_^j^*.

We define an *external* potential Φ_ext_ with associated *external* electric field **E**_ext_ ≔ −∇Φ_ext_, which is not limited by the constraint that Φ_ext_ tends to zero at infinity. We consider the external potential to be harmonic, i.e., ΔΦ_ext_ = 0, so that the charges creating the external field are not considered within the system. Furthermore, the electric field **E**_ext_ is not restricted to be *uniform*. Finally, we assume that the system of dielectric particles does not affect the external field **E**_ext_, for instance, through polarization, which justifies the use of our terminology “external”.

Our aim is to determine the total surface charge on each dielectric particle after taking into account both the free charge σ_f_, as well as the bound charges resulting from polarization effects due to the presence of charged neighboring particles and the effects of an external electric field. Using the total surface charge, we are able to deduce other physical quantities of interest such as the electrostatic forces and energy resulting from the interaction of *N* charged dielectric spheres both with each other and with an external electric field.

In order to determine the total surface charge, we first derive equations governing the *total electrostatic potential*. We show that the total electrostatic potential can be used to deduce the required total surface charge, as well as the subsequent physical quantities of interest. The main challenges in achieving our aim are to work with the singular nature of the point-charges σ_p_ and the external potential Φ_ext_, which does not decay to zero at infinity.

### 2.1 Formulation Based on Partial Differential Equations

The problem of the electrostatic interaction between *N* charged dielectric spheres can be described by a partial differential equation (PDE)- based transmission problem. To this end, we define the *total* potential (Φ_tot_) and the corresponding *total* electric field (**E_tot_**):
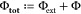

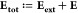
where **E** is the *perturbation* of **E_ext_**, which is due to the presence of dielectric charged particles, and Φ is the corresponding perturbation potential, such that **E** = −∇Φ. Standard arguments from the theory of electrostatics in dielectric media imply that the total potential Φ_tot_ satisfies the following transmission problem:
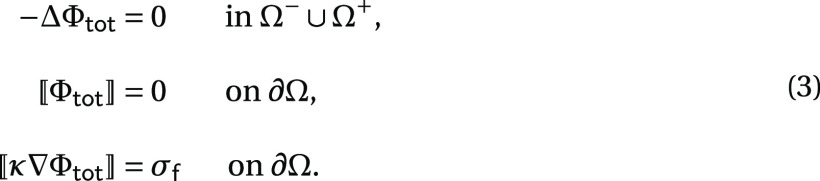
3Here, κ is the dielectric function, which takes the value of κ_*i*_ on the spherical particle Ω_*i*_ and κ_0_ on Ω^+^ (medium), and 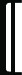
Φ_tot_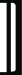 and 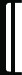
κ∇Φ_tot_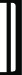 are jump discontinuities defined by
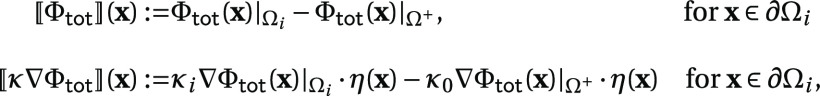
U1where η(**x**) is the normal unit vector at **x** ∈ ∂Ω pointing toward the exterior of the particles.

Generally, eq 3 is ill-posed as can be seen, for instance, by observing that if σ_f_ ≡ 0, then any constant function Φ_tot_ will satisfy this equation. In order to obtain the correct total potential Φ_tot_, we make use of the relation Φ_tot_ = Φ_ext_ + Φ and first derive a well-posed equation for the *perturbed* electrostatic potential Φ. Using decomposition (eq 1), elementary algebra shows that Φ satisfies the following transmission problem:
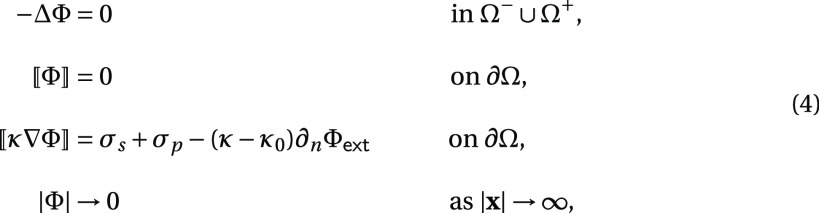
4where ∂_*n*_Φ_ext_ denotes the normal derivative of Φ_ext_ on the boundary ∂Ω.

PDEs similar to the transmission problem (eq 4) have previously been considered in the literature (see, e.g., refs (42 and 43)); however, the key novelty of eq 4 is the addition of contributions due to an external electric field and the presence of point charges on the surface of dielectric particles. These additional terms require significant modifications to earlier definitions^43−45^ of the electrostatic force and interaction energy for the *N*-body charged dielectric spheres, and they present additional challenges in the efficient numerical implementation.

In addition to the presence of the highly nonregular point-charge term σ_p_, another difficulty in solving the transmission problem (eq 4) is the fact that the equation is posed on the entire space . Indeed, since the potential Φ decays a priori only as |**x**|^–1^, a naive truncation of the computational domain in an effort to use classical algorithms, such as the finite element method, leads to significant errors. The usual approach to circumventing this problem is to appeal to the theory of integral equations and reformulate the transmission problem (eq 4) as a so-called “boundary integral equation” (BIE) posed on the interface ∂Ω. This is the subject of the next subsection.

### 2.2 Formulation Based on Boundary Integral Equations

In order to describe fully the integral equation-based approach to the problem of electrostatic interaction between charged dielectric spheres, we require additional notions. First, we define the single layer potential of some density ν, denoted , as a mapping with the property that
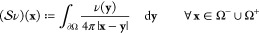
5It can be shown that, for any density ν,  is a harmonic function in Ω^–^ ∪ Ω^+^, which additionally satisfies the following jump conditions:
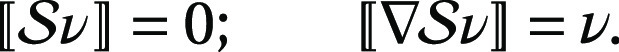
U2As a consequence, it is possible to consider a restriction of the single layer potential defined through eq 5 on the boundary ∂Ω and thereby define the so-called “single layer boundary” operator, denoted with  as the improper integral
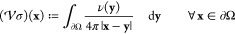
Note that, occasionally, it will be necessary to consider the “local” single layer potential and boundary operators defined on an individual sphere *i* ∈ {1, ..., *N*}. We will denote these as  and , respectively. Finally, let us remark that  is an invertible operator.

The surface electrostatic potential λ ≔ Φ|_∂Ω_ is now described by the following boundary integral equation:
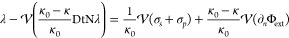
6Here, the notation DtN is used to denote the local Dirichlet-to-Neumann (DtN) map on the surface ∂Ω (see Section 1 of the Supporting Information).

An equivalent reformulation of the BIE (eq 6) for the induced surface charge can be achieved by applying  to both sides of the equation, and defining ν ≔ , which yields the following BIE:
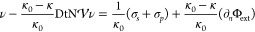
7In eq 7, the quantity of interest ν, which we call the induced surface charge, represents (up to a scaling factor) the total surface charge on each dielectric particle after taking into account both the free charge σ_f_ and the bound charge resulting from polarization effects, due to the presence of any remaining charged particles and the effect of an external electric field. More precisely,

- σ_f_ represents the free charge on each particle;
- σ_b_, which is defined as , represents the bound charge on each particle;
- κ_0_ν, which is defined as κ_0_ν = σ_f_ + σ_b_, represents the total surface charge on each particle.

A simple manipulation of eq 7 yields the following relation between the surface charge ν and the surface electrostatic potential λ:
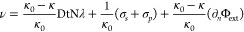
8Equation 8 implies that, once λ is known, the charge distribution ν can be computed using the purely local DtN map. We also remark here that the relation between the PDE (see eq 4) and the BIE (see eq 6) representations of the electrostatic potential can be clearly established since λ is simply the restriction (more precisely, the Dirichlet trace) of the electrostatic potential Φ on the boundary ∂Ω. Thus, for any point **x** ∈ Ω^–^ ∪ Ω^+^, we have Φ(**x**) =  = , and we therefore also have Φ_tot_(**x**) =  + .

As emphasized above, an important technical difficulty in the analysis of eq 6 is the presence of the low-regularity point-charge term σ_p_, which requires special treatment in the design of efficient numerical methods. The BIE (see eq 6) has previously been the subject of extensive analysis in a simpler case when surface point charges and the external field are absent, i.e., when σ_p_ ≡ 0 and Φ_ext_ ≡ 0. We first briefly summarize this methodology and explain how the BIE (see eq 6) can be solved in this simple case before turning our attention (in Section 3) to the more-complex problem of describing surface point charges and an external electric field.

### 2.3 Methodology in the Absence of Surface Point Charge and External Field

In the absence of both the point-charge contribution to the surface free charge and an external electric field, the boundary integral in eq 6 reads as
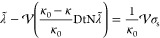
9Equation 9 is solved using a Galerkin discretization with an approximation space constructed from the span of finite linear combinations of local spherical harmonics on each sphere ∂Ω_*i*_ (an exact definitions of the spherical harmonics and the approximation space  can be found in Section 1 of the Supporting Information). More precisely, the Galerkin discretization of the BIE (9) reads as follows. Let  be a fixed discretization parameter, we seek the Galerkin solution , which satisfies, for all test functions,  the equation
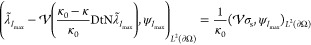
10

The Galerkin solution  and the test function  can be expanded as a finite linear combination of basis functions. This ansatz allows us to reduce the Galerkina discretization (eq 10) to a linear system of equations for the unknown expansion coefficients of . Thus, eq 10 yields the linear system
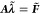
11where the solution matrix ***A*** and the vector  are defined as
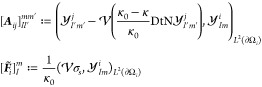
12where  denotes the spherical harmonic of degree  and order *m* on the sphere ∂Ω_*i*_ and the indices , , and . A more-detailed definition of  can be found in Section 1 of the Supporting Information, and a detailed explanation of how to compute the entries in the solution matrix ***A*** and vector  can be found in Lindgren et al.^42^ Here, we simply remark that, apart from the diagonal terms (*i* = *j*), computing the entries of the solution matrix and vector  requires evaluating a double integral on the unit sphere. This typically requires the use of numerical quadrature, for which purpose Lebedev grid points are used.

It is also possible to use a modification of the classical Fast Multipole Method (FMM) to speed up computation of the vector  and matrix-vector products involving the dense solution matrix **A**. Essentially, the FMM allows computing the action of the single-layer boundary operator  on an arbitrary element of the approximation space with linear scaling computational cost (with respect to *N*). Since the DtN map is a purely local operator (diagonal in the basis of local spherical harmonics), the solution matrix **A** does not need to be explicitly computed and stored, and its action on an arbitrary vector can be calculated with linear scaling cost. Further details on the FMM implementation can be found in Lindgren et al.^42^ Once the vector  has been computed and the procedure for applying the solution matrix **A** to an arbitrary vector in the approximation space is set up, the linear system (eq 11) can be solved using a Krylov subspace solver such as GMRES (see Bramas et al.^44^ for a detailed convergence analysis of GMRES, as applied to this linear system).

We can now turn our attention to calculation of the approximate electrostatic energy and force. The approximate electrostatic interaction energy of a dielectric *N*-body system is given by
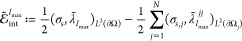
13where  and  is the approximate self-potential generated by the free charge σ_s,*j*_ on sphere ∂Ω_*j*_ in the absence of other spheres. More precisely, it is defined as the solution to the local Galerkin discretization
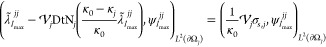
In the definition of the electrostatic interaction energy (eq 13), the first term can be interpreted as the *total* electrostatic energy of the system while the second term, involving the summation, can be seen as the *self* energy.

Next, we derive an expression for the approximate electrostatic forces. As a first step, if  denotes a solution to the Galerkin discretization (10) for a given free charge σ_s_, then we define the approximate induced surface charge  as the unique element of the approximation space  (defined in Section 1 of the Supporting Information) which satisfies
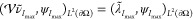
14In other words,  is simply an approximation of the exact induced surface charge ν, which physically represents the total surface charge on the dielectric spheres that includes polarization effects. Therefore, we use  to derive an expression for the approximate electrostatic force acting on the dielectric particles.

In practice,  is *not* determined using eq 14, which requires the computationally expensive inversion of the single-layer boundary operator V. Instead, a careful examination of the Galerkin discretization (eq 10) reveals that  satisfies the relation (cf, eq 8)
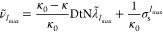
15where  is the best approximation of σ_s_ in the approximation space . Consequently, once the linear system (see eq 11) has been solved, only purely local operations involving the DtN operator are required to obtain .

The approximate electrostatic force acting on the dielectric particle is now given by
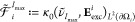
16 is the *i*-excluded electric field generated by the approximate induced surface charge , i.e., the vector field given by
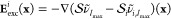
17where , and ∇ denotes the usual gradient, taken with respect to Cartesian coordinates. The *i*-excluded electric field  is the part of the total electric field generated by the approximate induced charge  that interacts with (i.e., exerts a net electrostatic force on) the dielectric particle Ω_*i*_. A description of how to practically compute  in the current boundary integral framework can be found in ref (45).

Consider the definitions of the approximate electrostatic interaction energy and force, given as eqs 13 and 16, respectively. A key result^45^ establishes that these are related by the identity
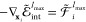
where  denotes the gradient taken with respect to the location of the center **x**_*i*_ of the sphere ∂Ω_*i*_.

The Galerkin nature of the method we present here allows for a precise mathematical analysis, in terms of accuracy, with respect to  and complexity with respect to *N*, which was previously discussed in Hassan et al.^43−45^ and also included the detailed description of the linear scaling of the method and the accuracy of predictions for the electrostatic energy and forces.^45^ However, the model is limited to the assumptions made at the beginning of Section 2.3, namely, it does not account for the presence of surface point charge and the effect of an external electric field. This extension and generalization is the subject of the following section.

### 2.4 Extension to Include an External Electric Field and Surface Point Charges

Turning our attention to the boundary integral, eq 6 is central to this study and describes the electrostatic interaction of dielectric spheres in the presence of both an external electric field and point-charge contributions to the free charge residing on the particle surface.

To begin, we define the external charge as σ_ext_≔ −(κ – κ_0_)∂_*n*_Φ_ext_, which is simply the external electric field contribution to the right-hand side of the boundary integral eq 6. The Galerkin discretization of the BIE (eq 6) can be written as
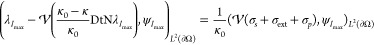
18

As stated previously, this Galerkin discretization (eq 18) yields a linear system of equations for the unknown local spherical harmonics expansion coefficients of  of the form
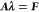
19where the solution matrix ***A*** is defined precisely as done previously through eq 12 and
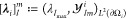
20for , , and . Determining the new vector ***F*** requires some additional work due to the presence of the point-charge term σ_p_. To this end, let **z**_*j*_ ∈ ∂Ω_*j*_ ⊂ ∂Ω. The definition of the single-layer boundary operator  implies that, for any , and, all **x** in ∂Ω with **x** ≠ **z**_*j*_, we have
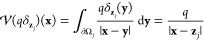
Hence,
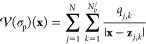
and, therefore, the vector ***F*** in eq 19 can be defined as
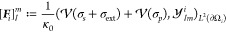
21Since the solution matrix **A** is exactly as before (see Section 2.3), one can use the same linear solver routine to approximate the solution to eq 19. Having solved the underlying linear system, we can now compute further (approximate) physical quantities of interest.

In computing the approximate electrostatic forces, if  denotes a solution to the Galerkin discretization (eq 18) for a given free charge σ_*f*_ = σ_s_ + σ_p_ and external electric field **E**_ext_, then we define, as in eq 14, the approximate induced surface charge  that generates the surface electrostatic potential  as the solution to
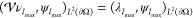
22In practice,  can be determined again using the following relation (c.f., eq 15), which can be deduced from the Galerkin discretization (eq 18):
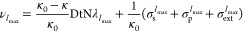
23where , , and  are the best approximations or projections (in the *L*^2^-sense) of σ_s_, σ_p_, and σ_ext_ in the approximation space  defined in Section 1 of the Supporting Information. The approximate net electrostatic force acting on the dielectric particle described by the open ball Ω_*i*_, *i* ∈ {1, ..., *N*} is now given by
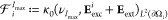
24where we remind the reader that  is the *i*-excluded electric field, which is defined analogously to eq 17. Let us remark here that  can practically be computed by adapting the procedure stated in ref (45) to the current setting of surface point charges and external electric field, which is not a difficult generalization.

In contrast to the definition of the electrostatic forces, the definition of the *electrostatic interaction energy* is not straightforward in the current setting. On the other hand, in the chemical literature, the net force acting on a given dielectric particle is frequently defined as the negative-sphere-centered gradient of the interaction energy. Keeping this relation in mind, the approximate electrostatic interaction energy of the system that corresponds to the approximate electrostatic force (see eq 24) is given by
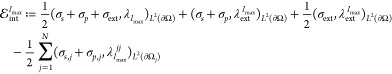
25where we denote ,  and we write  for the best approximation of λ_ext_ ≔ Φ_ext_|_∂Ω_ and  for the approximate self-potential on sphere ∂Ω_*j*_ in the absence of the external field **E**_ext_ and all other spheres. The latter quantity is formally defined as the solution to the local Galerkin discretization
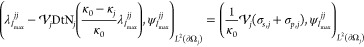


With the definitions of the approximate electrostatic interaction force and energy (described by eqs 24) and 25, respectively), we can demonstrate that the electrostatic forces are indeed realized as the negative sphere-centered gradients of the interaction energy.

**Theorem 2.1**. *Let**denote the approximate interaction energy and*, *denote the approximate electrostatic force acting on the dielectric particle* Ω_*i*_*as given by the definitions described by*eqs 25*and*24, *respectively. Then it holds that*
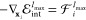
26*where**denotes the gradient taken with respect to the location of the center***x**_*i*_*of the sphere* ∂Ω_*i*_.

The proof of Theorem 2.1 can be found in Section 2 of the Supporting Information. Let us return to eq 25 that defines the electrostatic interaction energy of our system. Several comments are now in order.

First, it is important to emphasize that eq 25 includes both the energy due to the interaction between the dielectric particles themselves, as well as the energy arising from the interaction of particles with the external electric field.

Second, eq 25 has an interpretation in terms of the *total* and *self* electrostatic energies. Indeed, the combination of the first three terms in eq 25 can be interpreted as the *total* electrostatic energy of the system, while the fourth term, involving the summation, can be seen as the *self* electrostatic energy of the system. We emphasize that, because of the presence of the point-charge contribution σ_p_, both the total energy and the self-energies are infinite as in the case of fixed Coulomb point charges. However, when writing the interaction energy as
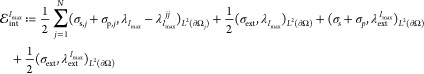
each of the terms is finite and, thus, the interaction energy is a well-defined quantity.

Finally, it is possible to rewrite eq 25 for the electrostatic interaction energy in a more physically intuitive form, in terms of the electric fields that appear in the PDE formulations (see eqs 3 and 4), leading to the following theorem.

**Theorem 2.2**. *Let λ_ext_denote the restriction of Φ_ext_to ∂Ω, and let λ denote the solution to the boundary integral eq 6 for a given free charge σ_f_ = σ_s_ + σ_p_ and external electric field **E**_ext_. Then, for any open ball**of radius r > 0, which is large enough to contain Ω^–^, the exact electrostatic interaction energy of the system, denoted**, satisfies the relation*
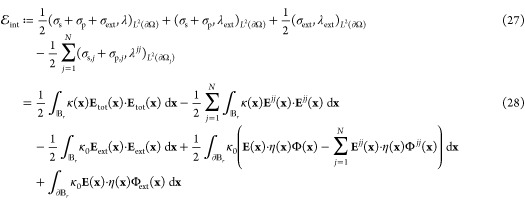
27Here, λ^*jj*^ is the exact self-potential only on sphere ∂Ω_*j*_ in the absence of an external field **E**_ext_ and all other spheres, and it is defined as the solution to the local BIE:
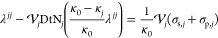
Moreover, **E**^*j j*^ and Φ^*jj*^ are the “self electric field” and “self electrostatic potential”, respectively, of the *j*th dielectric particle, i.e., the electric field and potential respectively produced only due to sphere ∂Ω_*j*_ in the absence of both the external field **E**_ext_ as well as the other spheres. The proof of Theorem 2.2 can be found in Section 2 of the Supporting Information.

The five terms in eq 28 which constitute  all have physical interpretations. Indeed, the first integral can be seen as the total electrostatic energy associated with an electric field **E**_tot_. The second integral can be interpreted as the self-energy associated with the free charge σ_*f*_ = σ_s_ + σ_p_ on the particle surface. The third term is the self-energy of the external electric field **E**_ext_. Finally, the last two terms can be interpreted as the boundary terms that, in general, may not vanish at infinity but yield an expression independent of the positions of the particles. Theorem 2.2 establishes that, in the exact case, i.e., when the discretization parameter *l*_max_ → ∞, the definition of the interaction energy, derived from the integral equation formalism and given by eq 25, coincides with the definition of the interaction energy (up to some additional boundary terms) in any open ball  that is large enough to contain Ω^–^, as derived from the PDE picture and given through eq 28.

Consider once again eq 24, which defines the net electrostatic force acting on dielectric particle Ω_*i*_. It is possible that one could be interested only in a portion of this electrostatic force *without* the so-called “self-force”. The self-force is the force that acts on the dielectric particle Ω_*i*_ in the absence of all other interacting particles but *still* in the presence of the external field **E**_ext_, i.e., the force that would act on the particle if it were the only one exposed to the external field. Mathematically, this new approximate net electrostatic force acting on the dielectric particle Ω_*i*_, *i* ∈ {1, ..., *N*} is given by the expression
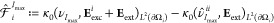
29where  is the total surface charge (including polarization effects) on ∂Ω_*i*_ in the absence of all other interacting particles but in the presence of the external electric field. Mathematically (c.f., eq 23),
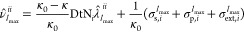
where  is the solution to the local Galerkin discretization
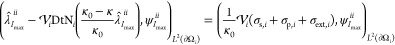
Corresponding to the approximate net electrostatic force given by eq 29, we have the following approximate interaction energy:
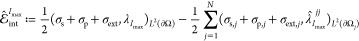
30

The force (see eq 29) subtracts the force that each single particle would be exposed to due to the external field in the absence of the other particles. The corresponding energy expression (eq 30) is then such that the force (eq 29) equals minus the sphere-centered gradients of the energy (30) following similar arguments as used in the proof of Theorem 2.1.

## 3 Case Studies and Discussion

**Figure 1:**
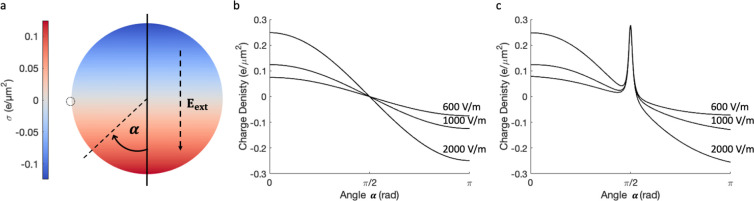
(a) Surface charge density on a neutral dielectric particle (κ = 10, *r* = 5 μm) placed in an external electrical field of **E**_**ext**_ = 1000 V/m. (b) Surface charge density on the neutral particle shown in panel (a), calculated at different external electrical field strengths (**E**_**ext**_ = 600, 1000, and 2000 V/m). (c) Surface charge density on the particle described in panel (a) with a model surface point charge of 0.2*e* placed at α = π/2, as indicated by a small dotted circle.

In this section, we benchmark the developed methodology starting with a single particle in the external field. When a uniform external electrical field **E**_ext_ is applied to a dielectric particle, redistribution of the surface charge creates a dipole aligned in the direction of the applied field. This effect is illustrated in Figure 1a where, for a neutral particle, the calculated variation in the surface charge density is shown for **E**_**ext**_ = 1000 V/m.

The dipole induced by the applied field is defined classically as^54^
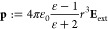
31where *r* is the particle radius, ϵ_0_ is the permittivity of free space, and ϵ is the relative permittivity of the particle, with respect to the medium (ϵ = κ/κ_0_). The dipole (see eq 31) can be represented by the surface charge distribution as
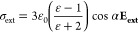
32A charged particle would also experience a force acting in the direction of the applied field,^54^ and, in the case of an inhomogeneous distribution of free surface charge, the particle will rotate to minimize the interaction energy with the field.^55^Figure 1c shows the distribution of the surface charge density, as a function of the angle α defined in Figure 1a. These calculations were completed using a sufficient value of the discretization parameter *l*_max_ to achieve the convergence of the interaction energy to the eighth decimal place. The value of *l*_max_ was evaluated for each study: particles with a surface point-charge (Figure 1c) required at least an approximation with *l*_max_ = 40 to achieve convergence (visually in the plots) at all angles α (a value of *l*_max_ = 45 was finally used with 3074 Lebedev integration points), while uniformly charged particles placed in an electrical field required at least *l*_max_ = 30 (and *l*_max_ = 35 was finally used with 1730 Lebedev integration points).

The interaction energy between two fixed dipoles, as defined by eq 31, is given by
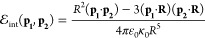
where *R* is the separation distance between their centers denoted by the vector **R**. It is convenient to express the direction of the dipoles **p**_**1**_ and **p**_**2**_, with respect to the vector **R**, using polar coordinates, such that
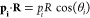
and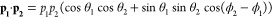 can then be rewritten as
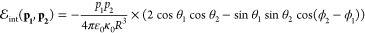
33

### 3.1 Separation-Dependent Particle Polarization

**Figure 2:**
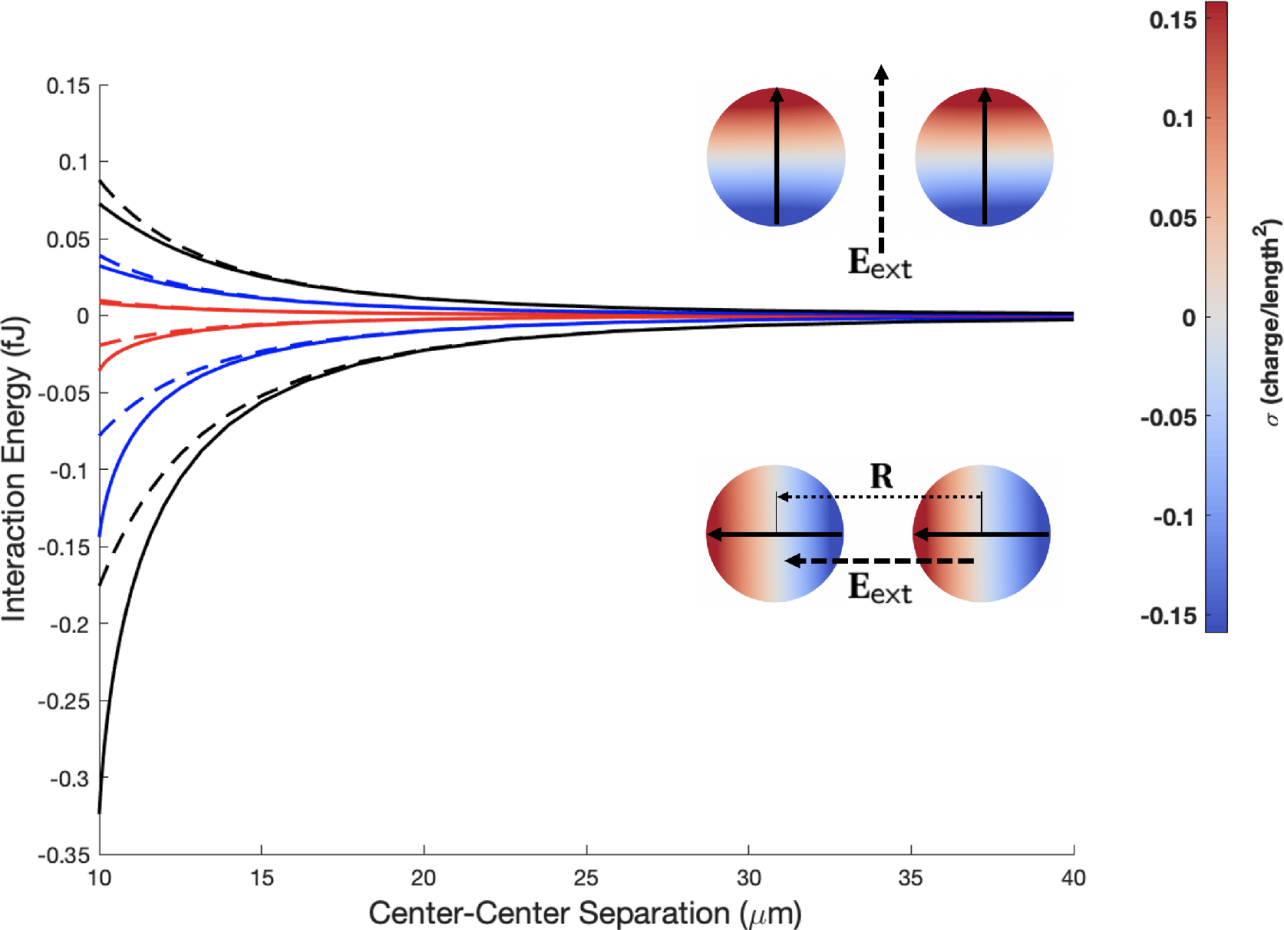
Interaction energy between two neutral dielectric particles (*r*_1_ = *r*_2_ = 5 μm and κ_1_ = κ_2_ = 10) in an applied electric field, as a function of the separation distance. Dashed line represents an approximation of two fixed dipoles, as defined by eq 33; solid line represents the calculation using eq 25, taking into account the separation-dependent particle polarization. The strength of the applied electric field is 100 kV/m (red), 200 kV/m (blue), and 300 kV/m (black). The interaction occurs in a vacuum, i.e., κ_0_ = 1.

At short separation distances, we note a significant difference in the accuracy between the approximation of two fixed dipoles (eq 33) and the model derived here, which takes into account the separation-dependent particle polarization.^57^Figure 2 shows the calculated interaction energy between two neutral particles of identical size and composition (*r*_1_ = *r*_2_ = 5 μm and κ_1_ = κ_2_ = 10) exposed in vacuum to a uniform external electric field of varied strength. This classical result^54^ is reproduced by the dashed lines in Figure 2 for three different values of the external electric field strength. When the dipoles are aligned with the vector **R**, i.e., when sin θ_1_ = sin θ_2_ = 0 and cos θ_1_ = cos θ_2_ = 1 (or −1), their interaction is attractive, i.e.,  = . If the dipoles are aligned perpendicular to vector **R**, then sin θ_1_ = sin θ_2_ = 1 (or −1), cos θ_1_ = cos θ_2_ = 0, and the resulting interaction is repulsive with  = , which is exactly a factor of 2 smaller in absolute value than  and of opposite sign. In both cases, the interaction energy decays as 1/*R*^3^, and if the field strength is halved, the interaction energy is reduced by a factor of 4.

Dielectric particles immersed in an external electric field also experience additional attractive forces at short separation distances due to induced multipolar interactions, which are taken into account in eq 25. As Figure 2 shows, these induced attractions are much stronger in the case of , because of the close proximity of regions of surface charge density of opposite sign residing on neighboring particles. The polarizing effects of surface charge become more significant at separation distances comparable to the size of the particles. In the case of attraction, the interaction energy between particles can be twice as large as that predicted by the approximation of fixed dipoles (eq 33). Consequently, at short separation distances, a quantitatively accurate account of the interaction energy (and the force) can only be achieved through a realistic description of surface charge polarization, i.e., a description beyond the induced dipole *l*_max_ = 1 approximation as we describe here, where, in the case of Figure 2, *l*_max_ = 30 with 1454 Lebedev integration points was used.

**Figure 3:**
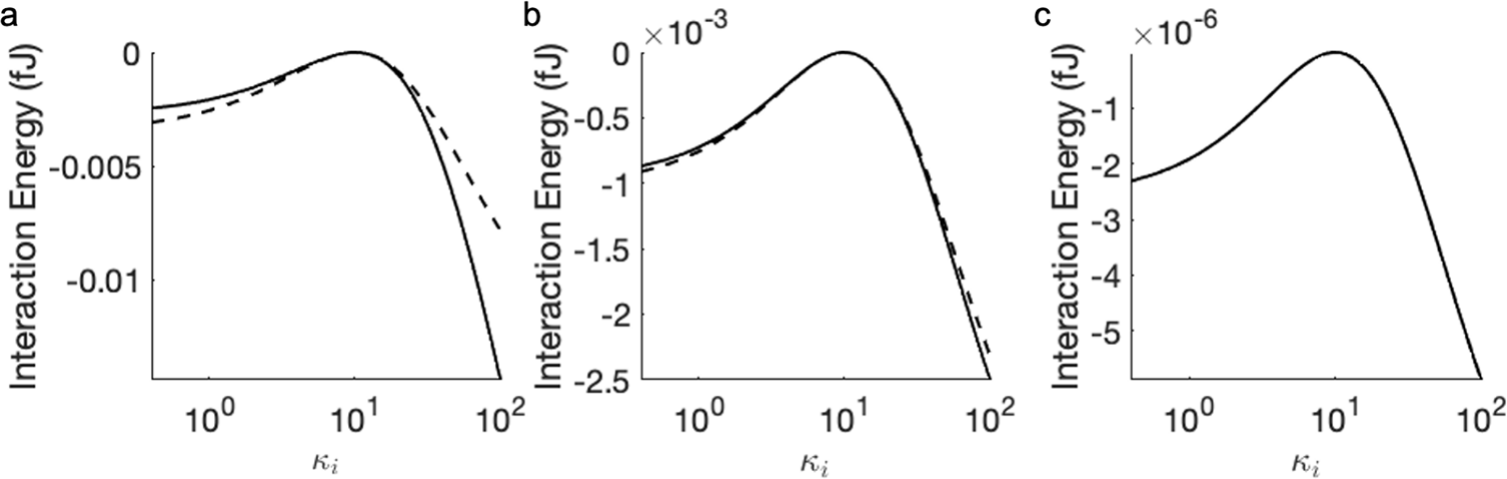
Interaction energy between two neutral particles (*r*_1_ = *r*_2_ = 5 μm) in an external electric field of 200 kV/m as a function of their dielectric constant. Dashed line represents the approximation of two fixed dipoles as defined by eq 33; solid line represents the calculation using eq 25. The surface-to-surface separation distance is (a) 10^–3^ μm, (b) 5 μm, and (c) 100 μm. The interaction happens in a medium with κ_0_ = 10. Note the change of scale along the *y*-axis.

**Figure 4:**
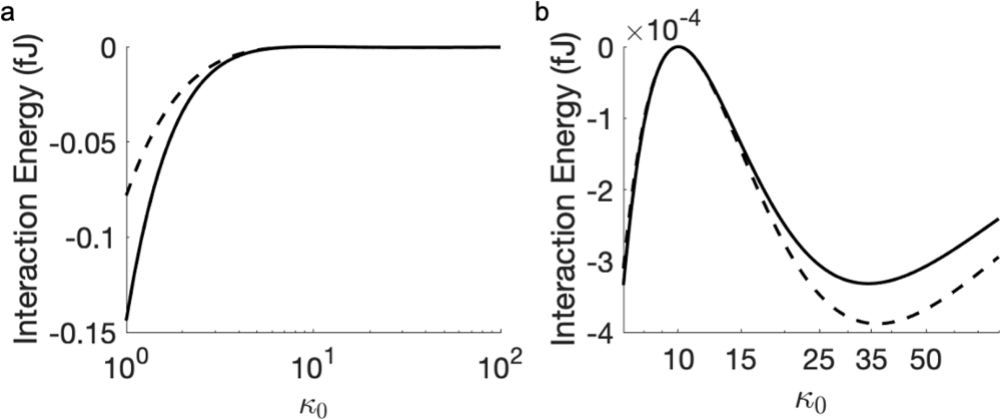
Interaction energy between two neutral particles (*r*_1_ = *r*_2_ = 5 μm and κ_1_ = κ_2_ = 10) in an external electric field of 200 kV/m as a function of the dielectric constant of medium: (a) κ_0_ ranging from 1 (vacuum) to 100; (b) expansion of the region for κ_0_ values between 10 and 45, highlighting minor extrema. Dashed line represents the approximation of two fixed dipoles, as defined by eq 33; solid line represents the calculation using eq 25. The surface-to-surface separation is 10^–3^ μm.

The nature of the attraction at short separations is also critically influenced by polarization of the medium, as shown in Figures 3 and 4. When the dielectric constant of the medium κ_0_ is greater than that of the particles κ_*i*_, shielding by the medium reduces the strength of the attractive interaction between particles. Figure 3a shows the most pronounced case of such a shielding effect at 10^–3^ μm surface-to-surface separation. At a large separation, as shown in Figure 3c, the shielding effect becomes negligible. When κ_0_ < κ_*i*_, the interaction is much stronger when particle polarization is taken into account, as confirmed in Figures 3a and 4a, and also in Figure 2. Figure 4 supplements these observations with calculations of the interparticle interaction energy for a large range of values of the dielectric constant of the medium—from 1 (vacuum) to 100. The simulations in both Figures 3 and 4 required spherical harmonics of the 30th degree (i.e., *l*_max_ = 30) with 1454 Lebedev integration points for the evaluation of eq 30.

### 3.2 Angular Dependence of Particle Interactions in an External Electric Field

**Figure 5:**
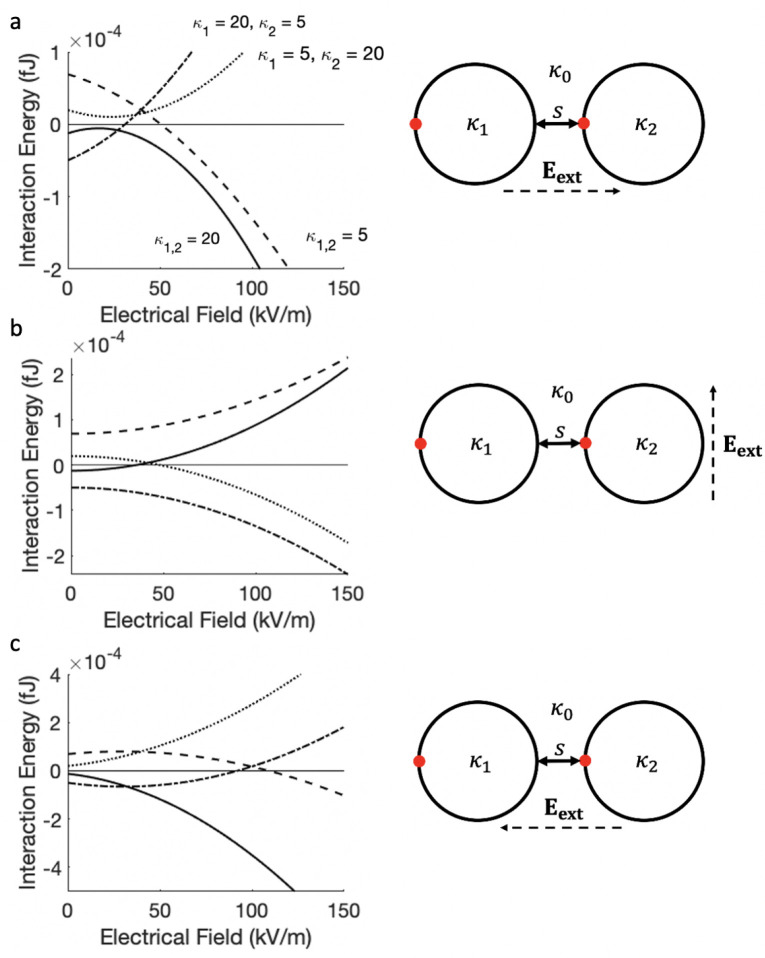
Interaction energy between two dielectric particles (*r*_1_ = *r*_2_ = 5 μm) containing a surface point charge of 50*e*, as a function of the strength of the applied external field: κ_1_ = κ_2_ = 20 (solid line), κ_1_ = κ_2_ = 5 (dashed line), κ_1_ = 20 and κ_2_ = 5 (dotted line), and κ_1_ = 5 and κ_2_ = 20 (dot-dashed line). The interaction occurs in a dielectric medium with κ_0_ = 10 at the surface-to-surface separation of 10^–3^ μm. Illustrations alongside each graph show the orientation of the external electric field parallel with (panels (a) and (c)) and perpendicular to (panel (b)) the alignment of the interacting particles.

Many chemical problems involving, for example, the adsorption of ions and protonation or deprotonation of functional groups on surfaces, require consideration of particles with an inhomogeneous distribution of surface charge, where the interaction is also dependent on the orientation of the particles. The special case of a neutral surface containing a point charge has been discussed in Filippov et al.,^26^ where the four extreme orientations of two point surface charges were considered in several different chemical scenarios; this work^26^ is in excellent agreement with the method presented here. For the general case, κ_*i*_ > κ_0_, the orientation of the particles shown in Figure 5 is the most attractive scenario in the absence of an external electric field. Furthermore, an inhomogeneous surface charge distribution, such as a point charge placed on a neutral sphere, breaks the axial symmetry (except for a few specific cases), thus presenting a more complex system.

**Figure 6:**
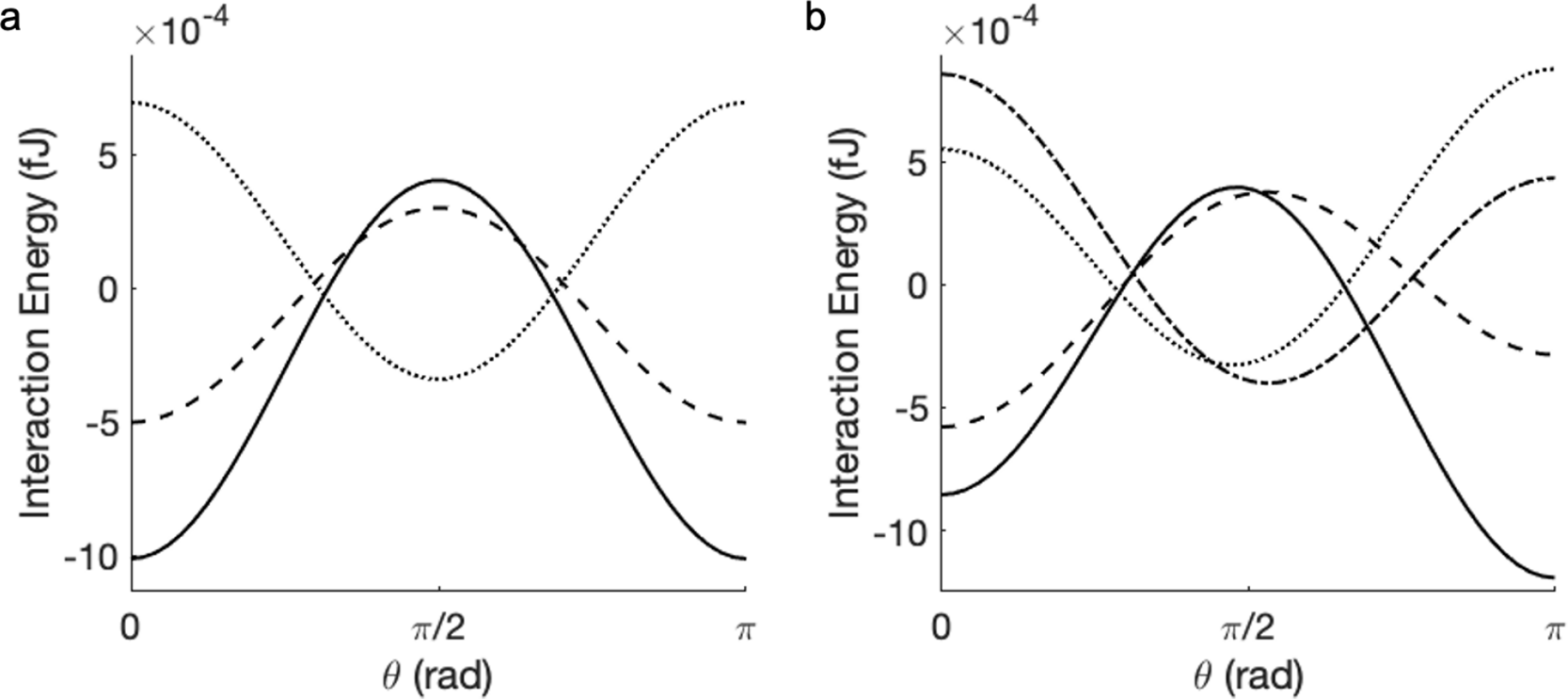
Interaction energy between two particles (*r*_1_ = *r*_2_ = 5 μm) in an external electric field of 200 kV/m as a function of the angle of the field rotation: (a) neutral dielectric particles and (b) dielectric particles with a point surface charge of 50*e*, as shown in Figure 5. Dashed line: κ_1_ = κ_2_ = 5; solid line: κ_1_ = κ_2_ = 20; dot-dashed line: κ_1_ = 20, κ_2_ = 5; dotted line: κ_1_ = 5, κ_2_ = 20. The interaction occurs in a medium with κ_0_ = 10 at the surface-to-surface separation of 10^–3^ μm. Note that, in the case of uniform surface charge distribution (a) the cases of κ_1_ = 20, κ_2_ = 5 and κ_1_ = 5, κ_2_ = 20 are identical.

As illustrated in Figure 2, the interaction between two particles in the presence of an external electric field has a strong directional dependence. If the strength of the applied electric field is high, the interaction between particles containing surface point charge follows the trends seen in Figure 2. In this case, the dominant contribution to the interaction energy/force comes from a field-induced dipole–dipole interaction. When both particles have the same dielectric constant (see the solid and dashed lines in Figures 5a–c), a strong attractive interaction occurs when the field is acting parallel to particle alignment (Figure 5a: θ = 0; and Figure 5c: θ = π); however, if κ_0_ > κ_*i*_ (dashed line), the dipole–dipole interaction is reduced due to the medium shielding effect. In Figure 5b, where the applied field acts in the direction perpendicular to particle alignment (θ = π/2), the interaction is driven by the repulsive dipole–dipole interaction. If κ_1_ < κ_0_ < κ_2_ (dot-dashed lines) or κ_2_ < κ_0_ < κ_1_ (dotted lines), the dominant dipole–dipole interaction is repulsive when the field is parallel to particle alignment, and it is attractive when the field is perpendicular to the particle alignment, as the dipoles point in opposite directions in the latter case. At smaller magnitudes of applied electric field, an additional contribution to the interaction energy from the surface point charges becomes significant, leading to more subtle effects. The strength of the interaction in this case is governed by the total surface charge represented by fixed point charges and induced surface charge. This behavior can be understood through eq 25 by realizing that  for weak external fields and  for strong external fields. However, as these studies refer to charged particles, the interaction energies in both Figures 5 and 6 are calculated via the evaluation of eq 30 in order to only study the interaction of the particles with each other.

The effect of orientating an applied external field on the interaction energy between two particles is detailed in Figure 6. With reference to Figure 2 for neutral particles, the most attractive interaction corresponds to the field orientation where the induced dipoles are aligned parallel with vector **R**. As the applied field rotates, the repulsive interaction between the regions of polarized charge of the same sign becomes stronger. At the angle corresponding to zero interaction energy, the opposing attractive and repulsive interactions cancel out. At the point of smallest separation, the exact value of this angle deviates from that predicted by eq 33 for two fixed-size dipoles as the induced polarization affects the interparticle interaction at all angles of rotation. Fixed dipole interactions go to zero at θ ≈ 0.96 rad, showing slight variations in the value of this angle if accounting for polarization effects. Polarization effects and the geometry of the problem are also responsible for the repulsion being smaller in magnitude than the attraction, which is expected given the results shown in Figure 2. For the case κ_2_ < κ_0_ < κ_1_, i.e., where one particle is less polarizable and the other more polarizable than the medium, the nature of the interparticle interaction in the applied electric field is inverted as shown by dot-dashed line in Figure 6a. When the applied field is parallel to vector **R**, the two like charged regions residing on the surface of the particles are closest, thus causing repulsion; when the field is perpendicular to the particle alignment (θ = π/2), the closest regions of high charge density are of opposite sign and attractive in nature. This result can be readily understood by an analysis of the field induced dipole given by eq 31 and by considering the resultant sign of the product **p**_**1**_·**p**_**2**_.

With the addition of a point charge to the surface of each particle, the interaction energy described by eq 25 is again driven by the total surface charge density having both  and  components. For the case of κ_1_ = κ_2_ = 20 polarization due to the point charge leads to a more attractive interaction at θ = π where the total surface charge at 10^–3^ μm surface-to-surface separation (*s*) increases due to the applied field; the interaction is less attractive at θ = 0 as the total charge at the closest *s* value decreases due to the field. The same reasoning can be applied to the case of κ_1_ = κ_2_ < κ_0_ but with the opposite overall effect. Similarly, in the case of κ_1_ < κ_0_ < κ_2_, the general shape can be attributed to the effects captured in Figure 6 (left) for neutral particles. The deviation in the interaction energy at θ = 0 and θ = π for the cases where κ_2_ ≠ κ_1_ is due to the polarization caused by the point charge on the surface of the neighboring particle.

In conclusion, the results presented in Figures 2–6 agree with the classical picture of interaction between two fixed-size dipoles, while showing variations in the strength of such interaction due to particle polarization, which are substantial when the interparticle separation is comparable to the size of the particles. A quantitative description of charged particles with inhomogeneous surface charge distributions interacting in an external electric field can be obtained readily using the formalism presented in Section 2.4.

### 3.3 Melting Ionic Colloidal Crystals in External Electric Fields

A better understanding of opposite-charge colloidal interactions could facilitate the controlled production of binary crystals with nanometer-sized constituent particles, which will ultimately find applications in advanced photonic materials.^56^ Leunissen et al.^52^ investigated the formation of apolar colloidal crystals consisting of poly(methyl methacrylate) (PMMA) particles with opposite, dissimilar charges and different sizes suspended in a density matching mixture of cyclohexyl bromide (CHB) and *cis*-decalin. The particle charge was regulated by the addition of tetrabutyl-ammonium bromide (TBAB) salt, which also controlled the Debye screening length. The authors^52^ reported that, for a broad range of particle sizes and charges, the PMMA particles formed body-centered cubic-type (cesium chloride) crystals, which could be reversibly destabilized by the application of an electric field.

The latter behavior can be explained by calculating the electrostatic force that charged particles experience in an external electric field. A force acting in the direction of the applied field creates a surface charge distribution different from that in the absence of the field (see Figure 1). When exposed to a sufficiently high electrical field, the dipolar nature of the surface charge distribution leads to repulsion between particles in the plane perpendicular to the direction of the field,^54^ behavior similar to that shown in Figure 2.

If the movement of surface charge causes a colloidal crystal to destabilize, then the energy required could be of significant practical interest, which would require the evaluation of eq 25; however, here, we evaluate eq 30. In the subsequent numerical results, the interaction energy between particles in the crystal only has the electrostatic component as described in Section 2.3 of this paper. We further assume a vanishingly small osmotic pressure, such that the crystals are self-supported by the cohesive energy; indeed, these were experimental conditions adopted by Leunissen et al.^52^

**Figure 7:**
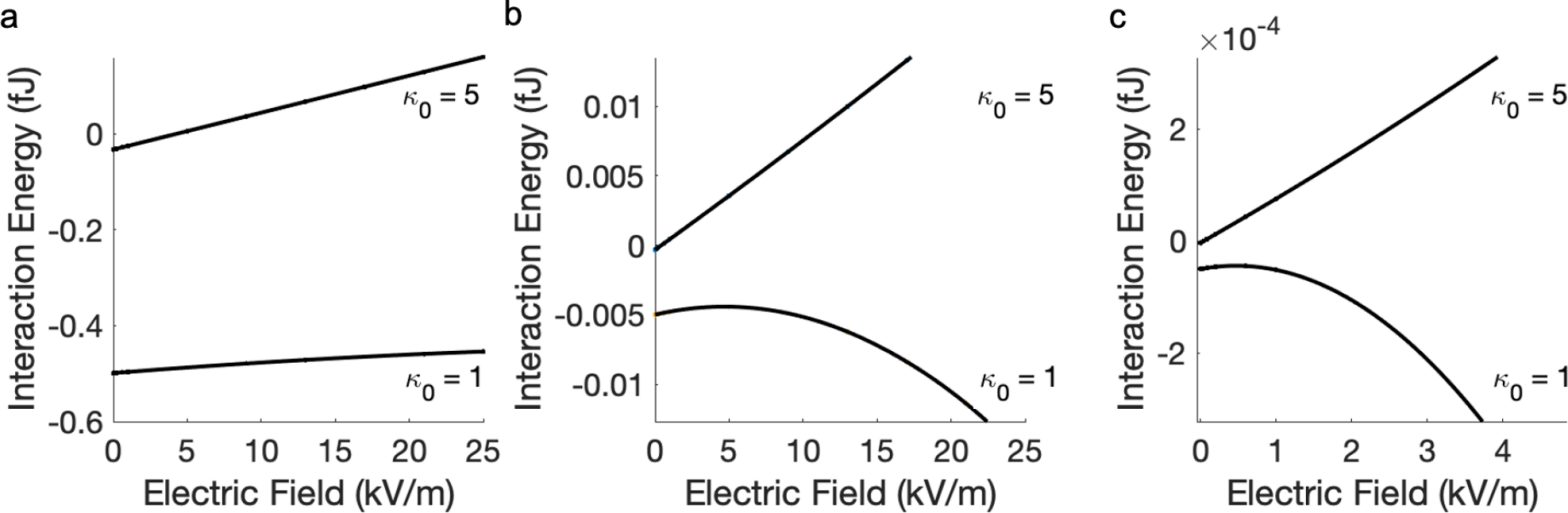
Interaction energy of PMMA colloidal crystal (κ_PMMA_ = 3, *r*_1_ = 1.08 μm, *r*_2_ = 0.99 μm, lattice parameter *a*_*l*_ = 2.4 μm), as a function of the applied electric field. The PMMA crystal is suspended under vacuum (κ_0_ = 1) and in solvent (κ_0_ = 5). The charge on PMMA particles is ±100*e* (a), ± 10*e* (b), ± 1*e* (c). In the absence of the external electric field, the interaction energy of the PMMA crystal is small but negative in all three cases.

Figure 7 presents the electrostatic energy of a PMMA crystal both under vacuum and in the presence of a solvent. The dielectric constant of the latter (κ_0_ = 5) matches that reported in experiments by Leunissen et al.^52^ The model crystal used in simulations contains 1024 particles, making it smaller than single crystals formed in experiments. Because of the negative value of the electrostatic interaction energy, the PMMA crystals in vacuum are predicted to be stable over a wide range of charge on the constituent particles. An interesting result from the calculations presented is that, under vacuum, the crystal can be stabilized even further with an increase of the strength of the applied field. This model also predicts that the PMMA crystal is stable in solvents in the absence of the applied electric field, but its structure can be destabilized by application of the field. Therefore, this model implies that if the solvent is more polarizable than the colloidal particles, then the crystal becomes unstable with increasing strength of the external field, as also seen in the experiments reported in Leunissen et al.,^52^ where κ_0_ = 5 was greater than κ_PMMA_ = 3.

**Figure 8:**
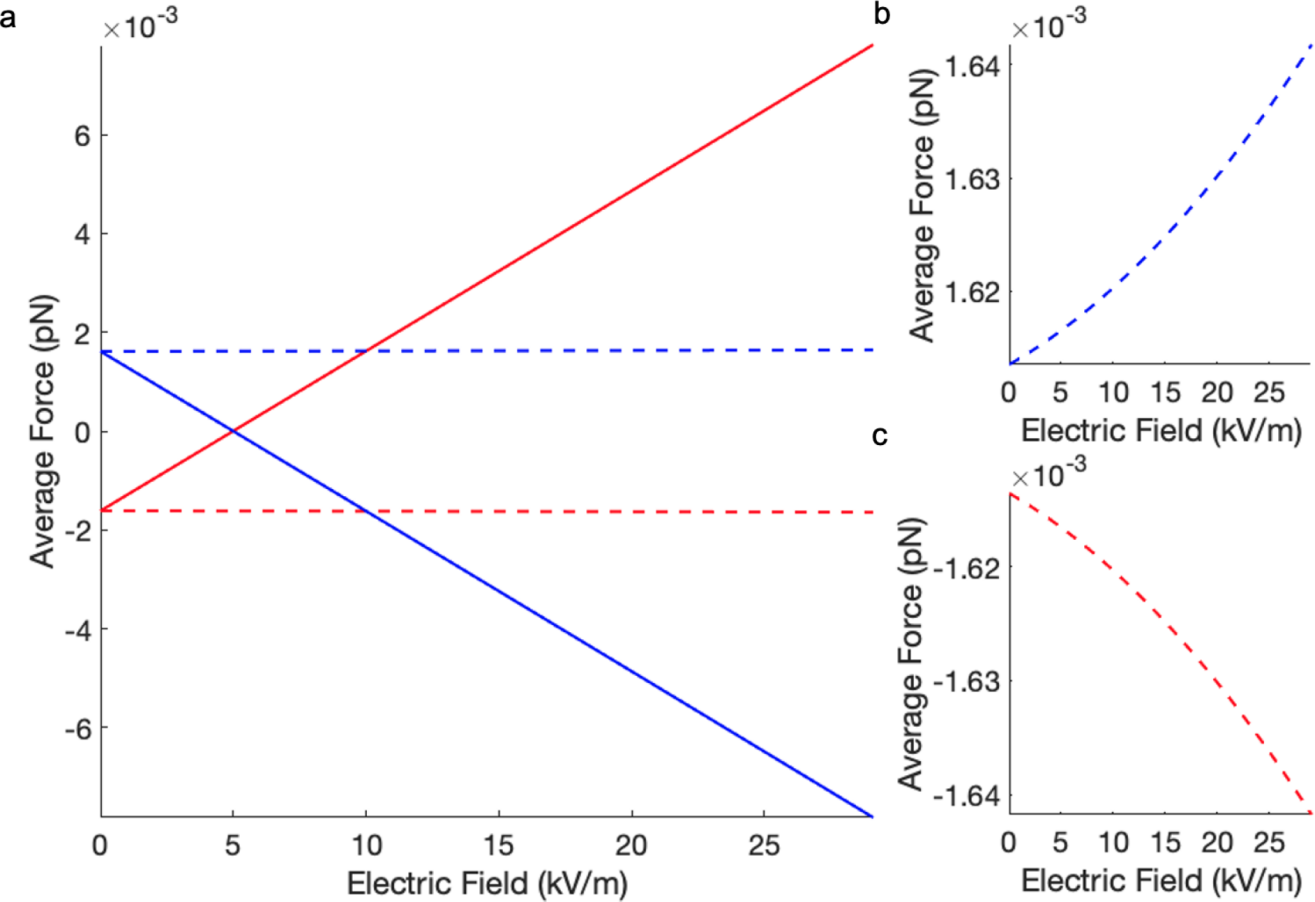
Average force acting on PMMA particles in the crystal (*z*_1,2_ = ±10*e*, *r*_1_ = 1.08 μm; *r*_2_ = 0.99 μm) suspended in solvent (κ_0_ = 5): (a) in the direction of the applied field (solid lines) and in the directions perpendicular to the field (dashed lines), (b) and (c) scaleup of the forces acting in the directions perpendicular to the field. The force on negative particles is depicted in blue and the force on positive particles is shown in red.

If the external field is switched on, the average electrostatic forces on oppositely charged particles act in opposite directions along the applied field, eventually causing the crystal structure to break apart and melt (see Figure 8). A more subtle change in the electrostatic force due to polarization occurs in directions perpendicular to the applied field. Figure 8a exhibits several interesting features, including the value of the field strength at which the average force on a particle in the direction of the applied field is zero and the point at which it crosses forces acting in the two directions perpendicular to the field. As Figure 8 shows, in the absence of an external field all three components of the net force on each particle have the same magnitude. At low field strength, the three components of the force are comparable in magnitude, and when the net force in the direction of the field is zero, the crystal particles still experience opposing and equal forces acting in the perpendicular directions (Figures 8b and 8c). Eventually, the direction of the force components parallel to the field change sign and become dominant with a further increase in the field strength, causing displacement of the oppositely charged particles in opposite directions along the field.

Experimental studies^52^ have reported observations of PMMA crystal melting through the application of an electrical field. At low values of the field strength (∼7 kV/m), a large CsCl-type crystal was found to be generally disordered. However, with the increase of the field strength to ∼20 kV/m, lane formation was observed. These findings can be explained using the calculations presented here (using spherical harmonics of the 13th degree with 266 Lebedev integration points). Disorder and melting of crystals occurs in the range of electric field values which are greater than the field strength corresponding to zero interaction energy in Figure 7 (positive interaction energies indicate unstable structures) but less than the value of the field at which the force components in Figure 8a are all equal in magnitude. Lane formation is observed at much higher fields, exceeding the value at which the force components in Figure 8a are equal. In this case, strong average forces acting on each particle, either in the direction of the field (positively charged particles) or antiparallel to the field (negatively charged particles), cause their spatial separation and lane formation.

### 3.4 Linear Scaling and Accuracy

**Figure 9:**
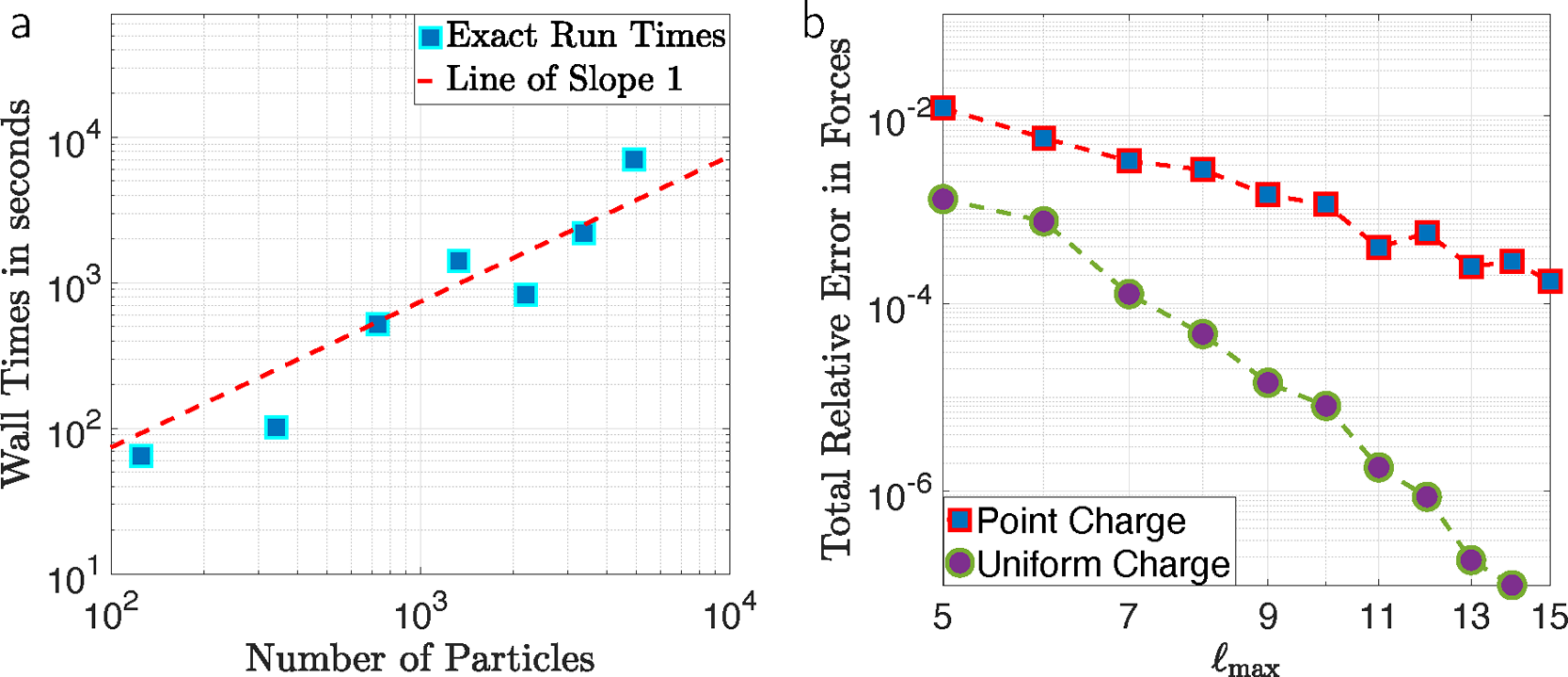
(a) Wall time for the computation of the energy and forces, with respect to the number of particles; (b) relative accuracy of the forces with respect to the discretization parameter *l*_max_ for systems with a free charge distribution consisting of uniform charge distributions and with point charges.

In these final numerical tests, we benchmark the performance of the FMM-based implementation of the method. We consider an arrangement of particles on a regular lattice of size *n* × *n* × *n*, for *n* = 5, 7, 9, ..., 17, thus ranging from 125 to 4913 particles. A uniform electric field of magnitude **E**_**ext**_ = 0.5 V/m along the *x*-axis is applied, and each particle contains a unit point charge at the north and south pole alternately. The radii and dielectric constants are alternating as well with values 3 and 2, and 50 and 300, respectively. The interaction occurs in a medium with κ_0_ = 10 and we use *l*_max_ = 10. The tolerance for the linear solver was set to a conservative threshold of 10^–10^. The results presented in Figure 9a were performed on a 2016 MacBook laptop with a 2.6 GHz Intel Core i7 processor and 16GB of 2133 MHz LPDDR3 memory. We observe that the execution time increases linearly. The change of regime between the first four data points and the last three is due to FMM adding one more layer in the tree structure.

We finish this section with a numerical study on the accuracy in calculating the forces, with respect to the discretization parameter . The tolerance for the linear solver was set to a very conservative threshold of 10^–13^. Figure 9b shows the relative error in the calculation of the force vector, with respect to a reference computation with large enough *l*_max_ values for the 5 × 5 × 5 lattice structure, for a uniform surface charge distribution, and with a surface point charge. In the presence of (singular) point charges, we observe an algebraic error decay with respect to *l*_max_, while the scenario with a uniform charge distribution shows superalgebraic convergence, which matches the theoretical result of exponential convergence for the case without an external field.^45^

## 4 Conclusion and Outlook

In this article, a theoretical framework based on boundary integral equations, suitable for computing the electrostatic interactions between particles undergoing mutual polarization, has been generalized to include two important physical effects: external, harmonic, nondecaying electric fields, and point-like charges localized on the particles’ surface. Analytical expressions for the interaction energy and the net electrostatic forces have been derived that allow computing these properties in linear scaling complexity, with respect to the number of interacting particles. The derived expressions ensure that the negative gradient of the interaction energy, with respect to the location of any given particle, equals precisely the net force acting on this particle. The longstanding computational challenges concerning singularities due to the presence of surface point-charges and a nondecaying external field, both of which formally lead to infinite energy if no special mathematical treatments are applied to the standard formalism, have been successfully resolved in this work. The developed formalism has been validated and tested using several numerical problems and applied to study the stability and melting of ionic colloidal suspensions in external electric fields.

The proposed methodology can be used in conjunction with other computational models to include the entropy associated with thermal effects and determine crystal stability at different temperatures, or it can be readily combined with estimations of the van der Waals forces where appropriate. In applications related to ionic crystals, however, the cohesive energy is dominated by Coulombic interactions, which are accurately described in the developed formalism. In this work, the net electrostatic force on each particle in a crystal has been computed by taking into account their separation-dependent polarization. This provides a rigorous and quantitatively accurate method, which allows one to explain the mechanisms underpinning electric-field-induced melting processes in ionic colloidal crystals and compare these predictions with existing experimental data. Approaches based on the fixed dipole approximation are not suitable in such cases, since they only describe the energetics of a chemical (or physical) process at long separations and are inaccurate when the interaction occur at distances comparable to the particle size.

Concerning future work, the nonuniform nature of the surface charge distribution implies that the interacting particles can no longer be seen as homogeneous. Consequently, rotational degrees of freedom must be taken into consideration in particle dynamics simulations based on the proposed formalism. While the methodology presented here can handle a single computation of the interaction energy (and force) for a given geometric configuration, the additional degrees of freedom must be updated during a time-dependent, dynamic simulation while respecting the torques acting on the particles. This will be the subject of a further contribution, which will provide a complete generalization of the proposed method.

## Supporting Information Available

Additional mathematical considerations, including details about mathematical assumptions, the properties of the mathematical objects used in this article, and a precise definition of the Galerkin approximation space that we use, as well as mathematical proofs of Theorems 2.1 and 2.2 (PDF)

## References

[ref1] Suehiro S.; Kimura T.; Tanaka M.; Takahashi S.; Mimura K.-i.; Kato K. Electrospray Deposition of 200 Oriented Regular-Assembly BaTiO3 Nanocrystal Films under an Electric Field. Langmuir 2019, 35, 5496–5500. 10.1021/acs.langmuir.8b03813. 30916558

[ref2] Mohammad T.; Bharti V.; Kumar V.; Mudgal S.; Dutta V. Spray coated europium doped PEDOT:PSS anode buffer layer for organic solar cell: The role of electric field during deposition. Org. Electron. 2019, 66, 242–248. 10.1016/j.orgel.2018.12.034.

[ref3] Wang F.; Martinuzzi R.; Zhu J. J.-X. Experimental study of particle trajectory in electrostatics powder coating process. Powder Technol. 2005, 150, 20–29. 10.1016/j.powtec.2004.12.001.

[ref4] Jedrusik M.; Swierczok A.; Teisseyre R. Experimental study of fly ash precipitation in a model electrostatic precipitator with discharge electrodes of different design. Powder Technol. 2003, 135–136, 295–301. 10.1016/j.powtec.2003.08.021.

[ref5] Buzea C.; Beydaghyan G.; Elliott C.; Robbie K. Control of power law scaling in the growth of silicon nanocolumn pseudo-regular arrays deposited by glancing angle deposition. Nanotechnology 2005, 16, 1986–1992. 10.1088/0957-4484/16/10/002. 20817960

[ref6] Walther A.; Müller A. Janus particles: Synthesis, self-assembly physical properties, and applications. Chem. Rev. 2013, 113, 5194–5261. 10.1021/cr300089t. 23557169

[ref7] Bormashenko E.; Bormashenko Y.; Pogreb R.; Gendelman O. Janus droplets: liquid marbles coated with dielectric/semiconductor particles. Langmuir 2011, 27, 7–10. 10.1021/la103653p. 21128604

[ref8] Gangwal S.; Cayre O.; Bazant M.; Velev O. Induced-charge electrophoresis of metallodielectric particles. Phys. Rev. Lett. 2008, 100, 058302 10.1103/PhysRevLett.100.058302. 18352441

[ref9] Dong L.; Huang J.; Yu K.; Gu G. Dielectric response of graded spherical particles of anisotropic materials. J. Appl. Phys. 2004, 95, 621–624. 10.1063/1.1633648.

[ref10] Chen J.; Zhang H.; Zheng X.; Cui H. Janus particle microshuttle: 1D directional self-propulsion modulated by AC electrical field. AIP Adv. 2014, 4, 031325 10.1063/1.4868373.

[ref11] van Blaaderen A.; Dijkstra M.; van Roij R.; Imhof A.; Kamp M.; Kwaadgras B.; Vissers T.; Liu B. Manipulating the self assembly of colloids in electric fields. Eur. Phys. J. Spec. Top. 2013, 222, 2895–2909. 10.1140/epjst/e2013-02065-0.

[ref12] Kalsin A.; Fialkowski M.; Paszewski M.; Smoukov S. K.; Bishop K. J. M.; Grzybowski B. A. Electrostatic self-assembly of binary nanoparticle crystals with a diamond-like lattice. Science 2006, 312, 420–424. 10.1126/science.1125124. 16497885

[ref13] Gast A.; Zukoski Z. Electrorheological fluids as colloidal suspensions. Adv. Colloid Interface Sci. 1989, 30, 153–202. 10.1016/0001-8686(89)80006-5.

[ref14] Parthasarathy M.; Klingenberg D. Electrorheology: mechanisms and models. Mater. Sci. Eng.. 1996, R17, 57–103. 10.1016/0927-796X(96)00191-X.

[ref15] Gasser U.; Weeks E.; Schofield A.; Pusey P. N.; Weitz D. A. Real-space imaging of nucleation and growth of colloidal crystallization. Science 2001, 292, 258–262. 10.1126/science.1058457. 11303095

[ref16] Yethiraj A.; Van Blaaderen A. A colloidal model system with an interaction tunable from hard sphere to soft and dipolar. Nature 2003, 421, 513–517. 10.1038/nature01328. 12556887

[ref17] Van Blaaderen A.; Ruel R.; Wiltzius P. Template-directed colloidal crystallization. Nature 1997, 385, 321–324. 10.1038/385321a0.

[ref18] Weeks E.; Crocker J.; Levitt A.; Schofield A.; Weitz D. Three-dimensional direct imaging of structural relaxation near the colloidal glass transition. Science 2000, 287, 627–632. 10.1126/science.287.5453.627. 10649991

[ref19] Aarts D. G. A. L.; Schmidt M.; Lekkerkerker H. N. W. Direct visual observation of thermal capillary waves. Science 2004, 304, 847–850. 10.1126/science.1097116. 15131300

[ref20] Baptiste J.; Williamson C.; Fox J.; Stace A.; Hassan M.; Braun S.; Stamm B.; Mann I.; Besley E. The influence of surface charge on the coalescence of ice and dust particles in the mesosphere and lower thermosphere. Atmos. Chem. Phys. 2021, 21, 8735 10.5194/acp-21-8735-2021.

[ref21] Lindgren E.; Stamm B.; Chan H.-K.; Maday Y.; Stace A.; Besley E. The effect of like-charge attraction on aerosol growth in the atmosphere of Titan. Icarus 2017, 291, 245–253. 10.1016/j.icarus.2016.12.013.

[ref22] Jan K.-M.; Chien S. Role of Surface Electric Charge in Red Blood Cell Interactions. J. Gen. Physiol. 1973, 61, 638–654. 10.1085/jgp.61.5.638. 4705641 PMC2203480

[ref23] Naderi Mehr F.; Grigoriev D.; Heaton R.; Baptiste J.; Stace A.; Puretskiy N.; Besley E.; Böker A. Self-assembly behavior of oppositely charged inverse bipatchy microcolloids. Small 2020, 16, 2000442 10.1002/smll.202000442. 32181972

[ref24] Bichoutskaia E.; Boatwright A.; Khachatourian A.; Stace A. Electrostatic analysis of the interactions between charged particles of dielectric materials. J. Chem. Phys. 2010, 133, 024105 10.1063/1.3457157. 20632746

[ref25] Derbenev I.; Filippov A.; Stace A.; Besley E. Electrostatic interactions between spheroidal dielectric particles. J. Chem. Phys. 2020, 152, 024121 10.1063/1.5129756. 31941309

[ref26] Filippov A.; Chen X.; Harris C.; Stace A.; Besley E. Interaction between particles with inhomogeneous surface charge distributions: Revising the Coulomb fission of dication molecular clusters. J. Chem. Phys. 2019, 151, 154113 10.1063/1.5119347. 31640356

[ref27] Lindgren E.; Derbenev I.; Khachatourian A.; Chan H.-K.; Stace A.; Besley E. Electrostatic self-assembly: Understanding the significance of the solvent. J. Chem. Theory Comput. 2018, 14, 905–915. 10.1021/acs.jctc.7b00647. 29251927

[ref28] Derbenev I.; Filippov A.; Stace A.; Besley E. Electrostatic interactions between charged dielectric particles in an electrolyte solution. J. Chem. Phys. 2016, 145, 084103 10.1063/1.4961091. 27586900

[ref29] Stace A. J.; Bichoutskaia E. Absolute electrostatic force between two charged particles in a low dielectric solvent. Soft Matter 2012, 8, 6210–6213. 10.1039/c2sm25602a.

[ref30] Derbenev I.; Filippov A.; Stace A.; Besley E. Electrostatic interactions between charged dielectric particles in an electrolyte solution: constant potential boundary conditions. Soft Matter 2018, 14, 5480–5487. 10.1039/C8SM01068D. 29926874

[ref31] Freed K. Perturbative many-body expansion for electrostatic energy and field for system of polarizable charged spherical ions in a dielectric medium. J. Chem. Phys. 2014, 141, 034115 10.1063/1.4890077. 25053309

[ref32] Barros K.; Sinkovits D.; Luijten E. Efficient and accurate simulation of dynamic dielectric objects. J. Chem. Phys. 2014, 140, 064903 10.1063/1.4863451. 24527936

[ref33] Barros K.; Luijten E. Dielectric effects in the self-assembly of binary colloidal aggregates. Phys. Rev. Lett. 2014, 113, 017801 10.1103/PhysRevLett.113.017801. 25032932

[ref34] Clercx H.; Bossis G. Many-body electrostatic interactions in electrorheological fluids. Phys. Rev. E 1993, 48, 2721 10.1103/PhysRevE.48.2721. 9960905

[ref35] Lotan I.; Head-Gordon T. An analytical electrostatic model for salt screened interactions between multiple proteins. J. Chem. Theory Comput. 2006, 2, 541–555. 10.1021/ct050263p. 26626662

[ref36] Linse P. Electrostatics in the presence of spherical dielectric discontinuities. J. Chem. Phys. 2008, 128, 214505 10.1063/1.2908077. 18537431

[ref37] Messina R. Image charges in spherical geometry: Application to colloidal systems. J. Chem. Phys. 2002, 117, 11062–11074. 10.1063/1.1521935.

[ref38] Xu Z. Electrostatic interaction in the presence of dielectric interfaces and polarization-induced like-charge attraction. Phys. Rev. E 2013, 87, 013307 10.1103/PhysRevE.87.013307. 23410460

[ref39] Qin J.; Li J.; Lee V.; Jaeger H.; de Pablo J.; Freed K. A theory of interactions between polarizable dielectric spheres. J. Colloid Interface Sci. 2016, 469, 237–241. 10.1016/j.jcis.2016.02.033. 26896771

[ref40] Qin J.; de Pablo J.; Freed K. Image method for induced surface charge from many-body system of dielectric spheres. J. Chem. Phys. 2016, 145, 124903 10.1063/1.4962832. 27782617

[ref41] Gan Z.; Jiang S.; Luijten E.; Xu Z. A hybrid method for systems of closely spaced dielectric spheres and ions. SIAM J. Sci. Comput. 2016, 38, B375–B395. 10.1137/15M105046X.

[ref42] Lindgren E.; Stace A.; Polack E.; Maday Y.; Stamm B.; Besley E. An integral equation approach to calculate electrostatic interactions in many-body dielectric systems. J. Comput. Phys. 2018, 371, 712–731. 10.1016/j.jcp.2018.06.015.

[ref43] Hassan M.; Stamm B. An Integral Equation Formulation of the *N*-Body Dielectric Spheres Problem. Part I: Numerical Analysis. ESAIM: Math. Model. Numer. Anal. 2021, 55, S65–S102. 10.1051/m2an/2020030.

[ref44] Bramas B.; Hassan M.; Stamm B. An Integral Equation Formulation of the *N*-Body Dielectric Spheres Problem. Part II: Complexity Analysis. ESAIM: Math. Model. Numer. Anal. 2021, 55, S625–S651. 10.1051/m2an/2020055.

[ref45] Hassan M.; Stamm B. A Linear Scaling in Accuracy Numerical Method for Computing the Electrostatic Forces in the *N*-Body Dielectric Spheres Problem. Commun. Comput. Phys. 2020, 29, 319–356. 10.4208/cicp.OA-2020-0090.

[ref46] Pusey P.; van Megen W. Phase behaviour of concentrated suspensions of nearly hard colloidal spheres. Nature 1986, 320, 340–342. 10.1038/320340a0.

[ref47] Kegel W.; Van Blaaderen A. Direct observation of dynamical heterogeneities in colloidal hard-sphere suspensions. Science 2000, 287, 290–293. 10.1126/science.287.5451.290. 10634780

[ref48] Pham K.; Puertas A.; Bergenholtz J.; Egelhaaf S.; Moussaïd A.; Pusey P.; Schofield A.; Cates M.; Fuchs M.; Poon W. Multiple glassy states in a simple model system. Science 2002, 296, 104–106. 10.1126/science.1068238. 11935020

[ref49] Kim Y.; Shah A.; Solomon M. Spatially and temporally reconfigurable assembly of colloidal crystals. Nat. Commun. 2014, 5, 3676 10.1038/ncomms4676. 24759549

[ref50] Habdas P.; Weeks E. R. Video microscopy of colloidal suspensions and colloidal crystals. Curr. Opin. Colloid Interface Sci. 2002, 7, 196–203. 10.1016/S1359-0294(02)00049-3.

[ref51] van Dommelen R.; Fanzio P.; Sasso L. Surface self-assembly of colloidal crystals for micro- and nano-patterning. Adv. Colloid Interface Sci. 2018, 251, 97–114. 10.1016/j.cis.2017.10.007. 29174673

[ref52] Leunissen M.; Christova C.; Hynninen A.-P.; Royall C. P.; Campbell A.; Imhof A.; Dijkstra M.; van Roij R.; Van Blaaderen A. Ionic colloidal crystals of oppositely charged particles. Nature 2005, 437, 235–240. 10.1038/nature03946. 16148929

[ref53] Gelinck G.; Huitema H.; van Veenendaal E.; Cantatore E.; Schrijnemakers L.; van der Putten J.; Geuns T.; Beenhakkers M.; Giesbers J.; Huisman B.-H.; Meijer E.; Benito E.; Touwslager F.; Marsman A.; van Rens B.; de Leeuw D. Flexible active-matrix displays and shift registers based on solution-processed organic transistors. Nat. Mater. 2004, 3, 106–110. 10.1038/nmat1061. 14743215

[ref54] Stone A. J. The Theory of Intermolecular Forces, 2nd Edition; Oxford University Press: Oxford, U.K., 2013.

[ref55] Feynman R. The Feynman Lectures on Physics. Vol. 2, Mainly Electromagnetism and Matter; Addison–Wesley: Reading, MA, 1964.

[ref56] Vermolen E.; Kuijk A.; Filion L.; Hermes M.; Thijssen J.; Dijkstra M.; Van Blaaderen A. Fabrication of large binary colloidal crystals with a NaCl structure. Proc. Natl. Acad. Sci. U. S. A. 2009, 106, 16063–16067. 10.1073/pnas.0900605106. 19805259 PMC2740731

[ref57] Baptiste J. Electrostatic Theory of Charged Particle Polarisation: Applications in Self-Assembly, Pharmaceutical and. Atmospheric Processes; Ph.D. Thesis, submitted to the University of Nottingham, University Park, U.K.

