# Supplementary material for: Manipulating Interactions between Dielectric Particles with Electric Fields: A General Electrostatic Many-Body Framework
Source: J Chem Theory Comput. 2022 Sep 8;18(10):6281–96. doi: 10.1021/acs.jctc.2c00008 (PMC9558380; doi:10.1021/acs.jctc.2c00008)
Supplement: Supplementary file 1 — ct2c00008_si_001.pdf [file ct2c00008_si_001.pdf]

Supporting Information

Manipulating interactions between dielectric particles with electric fields: A general electrostatic many-body framework

Muhammad Hassan,[†] Connor Williamson,[‡] Joshua Baptiste,[‡] Stefanie Braun,[¶] Anthony J. Stace,[‡] Elena Besley,[*,‡] and Benjamin Stamm[*,§]

†*Sorbonne Université, CNRS, Université de Paris, Laboratoire Jacques-Louis Lions (LJLL), F-75005 Paris, France*

‡*School of Chemistry, University of Nottingham, University Park, NG7 2RD, UK*

¶*Mathematics Department, RWTH Aachen University, Schinkelstr. 2, 52062 Aachen, Germany*

§*Institute of Applied Analysis and Numerical Simulation, University of Stuttgart, Pfaffenwaldring 57, 70569 Stuttgart, Germany*

E-mail: Elena.Besley@nottingham.ac.uk; best@ians.uni-stuttgart.de

1 Mathematical considerations

This section contains some additional mathematical considerations such as more details about our mathematical assumptions, the properties of the mathematical objects used in this article and a precise definition of the Galerkin approximation space that we use.

To begin with, we assume that the external harmonic potential we consider satifies $\Phi_{\text{ext}} \in H^1_{\text{loc}}(\mathbb{R}^3)$ with the associated *external* electric field $\mathbf{E}_{\text{ext}} := -\nabla\Phi_{\text{ext}} \in L^2_{\text{loc}}(\mathbb{R}^3)$, where $L^2_{\text{loc}}(\mathbb{R}^3)$ and $H^1_{\text{loc}}(\mathbb{R}^3)$ denote the spaces of locally square integrable functions and locally square integrable functions with locally square integrable first derivatives, respectively. Next, we emphasise that, as is common in the mathematical literature, the solution to the PDE (4), i.e., the perturbed electrostatic potential Φ, is typically understood as an element of the space $H^1(\Omega^-) \cup H^1(\Omega^+)$ and is therefore not, in general, continuous. Strictly speaking therefore, the transmission conditions in Equation (4) must be understood in the sense of so-called Dirichlet and Neumann traces in the Sobolev spaces $H^{\frac{1}{2}}(\partial\Omega)$ and $H^{-\frac{1}{2}}(\partial\Omega)$ respectively. A detailed description of trace operators and fractional Sobolev spaces is beyond the scope of this article and can, for instance, be found in.[1]

Concerning the mapping properties of the single layer potential and boundary operators, it can be shown that for any $s \in \mathbb{R}$, the mapping $\mathcal{S}$ extends as a bounded linear map from the Sobolev space $H^s(\partial\Omega)$ to $H^{s+3/2}_{\text{loc}}(\mathbb{R}^3)$ and the operator $\mathcal{V}$ extends as an *invertible*, bounded linear map from $H^s(\partial\Omega)$ to $H^{s+1}(\partial\Omega)$ (see, e.g.,[1] for a concise exposition on Sobolev spaces and for precise definitions and properties of the single layer potential). "Local" versions of the single layer potential and boundary operators which we have used frequently in this article are formally defined as follows: For each $i \in \{1, \ldots, N\}$, we have

$$
\begin{aligned}
(\mathcal{S}_i \nu_i)(\mathbf{x}) &:= \int_{\partial\Omega_i} \frac{\nu_i(\mathbf{y})}{4\pi|\mathbf{x}-\mathbf{y}|}\, d\mathbf{y}, \qquad \forall \mathbf{x} \in \Omega_i \cup \mathbb{R}^3 \setminus \overline{\Omega_i}, \quad \forall \nu_i \in H^s(\partial\Omega_i),\ s \in \mathbb{R},\\
(\mathcal{V}_i \nu_i)(\mathbf{x}) &:= \int_{\partial\Omega_i} \frac{\nu_i(\mathbf{y})}{4\pi|\mathbf{x}-\mathbf{y}|}\, d\mathbf{y}, \qquad \forall \mathbf{x} \in \partial\Omega_i, \qquad\qquad \forall \nu_i \in H^s(\partial\Omega_i),\ s \in \mathbb{R}.
\end{aligned}
$$

In addition, we have used extensively in this article, the so-called Dirichlet-to-Neumann map, denoted DtN. Mathematically, the map $\text{DtN}\colon H^s(\partial\Omega) \to H^{s-1}(\partial\Omega),\ s \in \mathbb{R}$ is defined as follows: Given some boundary function $\lambda \in H^s(\partial\Omega)$, let u_λ denote the harmonic extension of λ in Ω^-. Then $\text{DtN}\lambda \in H^{s-1}(\partial\Omega)$ is the normal derivative (more precisely, the Neumann trace) of u_λ on

the boundary $\partial\Omega$. We emphasise that in contrast to the single layer potential and boundary operator, the DtN map is a purely local operator, i.e., for any $\lambda \in H^s(\partial\Omega)$, $\mathrm{DtN}\lambda|_{\partial\Omega_i}$ depends only on $\lambda|_{\partial\Omega_i}$.

Concerning the regularity of solutions to the BIE (6), we recall from Equation (2) that the point-charge contribution σ_p to the free surface charge is assumed to be a linear combination of Dirac delta distributions. It is possible to show therefore that σ_p is an element of the Sobolev space $H^r(\partial\Omega)$ for every $r < -1$. In view of the regularising property of the single layer boundary operator $\mathcal{V}$, we can conclude that the right-hand side of the BIE (6) is, in general, an element of $H^r(\partial\Omega)$ for every $r < 0$. This implies in particular that solutions to the BIE (6) are not, in general, square integrable functions. On the other hand, we recall that $\sigma_s \in L^2(\partial\Omega)$ by assumption so that solutions to the BIE (9) can be readily understood as elements of the Sobolev space $H^1(\partial\Omega)$.

Finally, let us state the definition of the approximation space used in the proposed Galerkin discretisation.

Definition (Spherical Harmonics) For every integer $\ell \in \mathbb{N} \cup \{0\}$ and $m \in \{-\ell, \ldots, \ell\}$ we define $\mathcal{Y}_\ell^m \colon \mathbb{S}^2 \to \mathbb{R}$ as the real-valued L^2-orthonormal spherical harmonic of degree ℓ and order m on the unit sphere $\mathbb{S}^2$ (see[2] for a precise, constructive definition).

The set of spherical harmonics is dense in $L^2(\mathbb{S}^2)$ and is therefore well-suited for the choice of basis functions in the Galerkin discretisation of BIE (9).

Definition (Approximation Spaces) Let $\ell_{\max} \in \mathbb{N}$ be a discretisation parameter. First, on each sphere $\partial\Omega_i$, $i = 1, \ldots, N$ we define a local approximation space $W^{\ell_{\max}}(\partial\Omega_i)$ as

$$W^{\ell_{\max}}(\partial\Omega_i) := \left\{ u \colon \partial\Omega_i \to \mathbb{R} \quad \text{such that} \quad u(\mathbf{x}) = \sum_{\ell=0}^{\ell_{\max}} \sum_{m=-\ell}^{m=+\ell} [u]_\ell^m \mathcal{Y}_{\ell m}^i(\mathbf{x}) \quad \text{with} \quad [u]_\ell^m \in \mathbb{R} \right\},$$

where we introduced for notational convenience the basis functions $\mathscr{Y}_{\ell m}^{i} \colon \partial\Omega_i \to \mathbb{R}$ as

$$\mathscr{Y}_{\ell m}^{i}(\mathbf{x}) := \mathscr{Y}_{\ell}^{m}\left(\frac{\mathbf{x}-\mathbf{x}_i}{|\mathbf{x}-\mathbf{x}_i|}\right) \qquad \forall \mathbf{x} \in \partial\Omega_i.$$

Next, we define the global approximation space $W^{\ell_{\max}}$ as

$$W^{\ell_{\max}} := \Big\{u \colon \partial\Omega \to \mathbb{R} \text{ such that } \forall i \in \{1,\dots,N\} \colon u|_{\partial\Omega_i} \in W^{\ell_{\max}}(\partial\Omega_i)\Big\}.$$

2 Mathematical Proofs of Theorems 2.1 and 2.2

In this section we provide proofs of Theorems 2.1 and 2.2 from Section 2.4. For technical reasons, it is useful to begin with the proof of Theorem 2.2. This result shows that the definition of the interaction energy that we have provided in this article using quantities of interest from the integral equation (6) is consistent with the electric field-based definition of the interaction energy as derived directly from the PDEs (3) and (4). Throughout this section, we will use the notation and setting introduced in Sections 2.1, 2.2 and 2.4.

2.1 Proof of Theorem 2.2

Let $j \in \{1,\dots,N\}$ and let $\mathbb{B}_r$ be an open ball large enough so that $\Omega^- \subset \mathbb{B}_r$. We begin by defining precisely $\mathbf{E}^{jj}$, i.e., the electric field produced *only due to the sphere* $\partial\Omega_j$ in the absence of both the external field $\mathbf{E}_{\text{ext}}$ as well as the other spheres. Maxwell's equations imply that $\mathbf{E}^{jj} :=$

$-\nabla\Phi^{jj}$ where the self-potential Φ^{jj} satisfies the PDE (c.f., Equation (4))

$$
\begin{aligned}
-\Delta\Phi^{jj} &= 0 && \text{in } \Omega_j \cup \mathbb{R}^3 \setminus \overline{\Omega_j} \\
[\![\Phi^{jj}]\!] &= 0 && \text{on } \partial\Omega_j, \\
[\![\kappa\nabla\Phi^{jj}]\!] &= \sigma_{s,j} + \sigma_{p,j} && \text{on } \partial\Omega_j, \\
|\Phi^{jj}| &\to 0 && \text{as } |\mathbf{x}| \to \infty,
\end{aligned}
\tag{1}
$$

where we remind the reader that $\sigma_{s,j} := \sigma_s|_{\partial\Omega_j}$ and $\sigma_{p,j} := \sigma_p|_{\partial\Omega_j}$.

Next, to aid the subsequent exposition, we define the auxiliary quantity

$$
\mathscr{E}^r_{\text{PDE,int}} := \int_{\mathbb{B}_r} \kappa(\mathbf{x})\mathbf{E}_{\text{tot}}(\mathbf{x}) \cdot \mathbf{E}_{\text{tot}}(\mathbf{x})\, d\mathbf{x} - \sum_{j=1}^{N} \int_{\mathbb{B}_r} \kappa(\mathbf{x})\mathbf{E}^{jj}(\mathbf{x}) \cdot \mathbf{E}^{jj}(\mathbf{x})\, d\mathbf{x} - \int_{\mathbb{B}_r} \kappa_0 \mathbf{E}_{\text{ext}}(\mathbf{x}) \cdot \mathbf{E}_{\text{ext}}(\mathbf{x})\, d\mathbf{x}.
\tag{2}
$$

We may now use simple algebra and the fact that $\Phi_{\text{tot}} = \Phi + \Phi_{\text{ext}}$ (see Section 2.1) to deduce that

$$
\begin{aligned}
\mathscr{E}^r_{\text{PDE,int}} = &\int_{\mathbb{B}_r} \kappa(\mathbf{x}) \left|\nabla\Phi(\mathbf{x})\right|^2 d\mathbf{x} + 2\int_{\mathbb{B}_r} \kappa(\mathbf{x}) \nabla\Phi(\mathbf{x}) \cdot \nabla\Phi_{\text{ext}}(\mathbf{x})\, d\mathbf{x} \\
&- \sum_{j=1}^{N} \int_{\mathbb{B}_r} \kappa(\mathbf{x}) \left|\nabla\Phi^{jj}(\mathbf{x})\right|^2 d\mathbf{x} + \int_{\mathbb{B}_r} (\kappa(\mathbf{x}) - \kappa_0) \left|\nabla\Phi_{\text{ext}}(\mathbf{x})\right|^2 d\mathbf{x}.
\end{aligned}
$$

Next, we recall from the PDEs (3) and (4) that Φ is harmonic on $\Omega^- \cup \Omega^+$, Φ_{ext} is harmonic on $\mathbb{R}^3$, and Φ^{jj} is harmonic on $\Omega_j \cup (\mathbb{R}^3 \setminus \overline{\Omega_j})$. Therefore we can appeal to Green's first identity

to simplify the above integrals as

$$
\begin{aligned}
\int_{\mathbb{B}_r} \kappa(\mathbf{x}) \left|\nabla\Phi(\mathbf{x})\right|^2 d\mathbf{x} &= \int_{\partial\Omega} [\![\kappa\nabla\Phi]\!](\mathbf{x})\Phi(\mathbf{x})\, d\mathbf{x} + \int_{\partial\mathbb{B}_r} \kappa_0 \partial_n\Phi(\mathbf{x})\Phi(\mathbf{x})\, d\mathbf{x},\\
2\int_{\mathbb{B}_r} \kappa(\mathbf{x})\nabla\Phi_{\text{ext}}(\mathbf{x})\cdot\nabla\Phi(\mathbf{x}) &= 2\int_{\partial\Omega} [\![\kappa\nabla\Phi]\!](\mathbf{x})\Phi_{\text{ext}}(\mathbf{x})\, d\mathbf{x} + 2\int_{\partial\mathbb{B}_r} \kappa_0 \partial_n\Phi(\mathbf{x})\Phi_{\text{ext}}(\mathbf{x})\, d\mathbf{x},\\
\int_{\mathbb{B}_r} \sum_{j=1}^{N} \kappa(\mathbf{x}) \left|\nabla\Phi^{jj}(\mathbf{x})\right|^2 d\mathbf{x} &= \sum_{j=1}^{N} \int_{\partial\Omega_j} (\kappa_j - \kappa_0)\partial_n\Phi^{jj}(\mathbf{x})\Phi^{jj}(\mathbf{x})\, d\mathbf{x} + \sum_{j=1}^{N} \int_{\partial\mathbb{B}_r} \kappa_0\partial_n\Phi^{jj}(\mathbf{x})\Phi^{jj}(\mathbf{x})\, d\mathbf{x},\\
\int_{\mathbb{B}_r} (\kappa(\mathbf{x}) - \kappa_0)\left|\nabla\Phi_{\text{ext}}(\mathbf{x})\right|^2 d\mathbf{x} &= \int_{\partial\Omega} (\kappa(\mathbf{x}) - \kappa_0)\partial_n\Phi_{\text{ext}}(\mathbf{x})\Phi_{\text{ext}}(\mathbf{x})\, d\mathbf{x}.
\end{aligned}
$$

Recalling the interface conditions from the PDEs (4) and (1), we can further simplify several of these integral as

$$
\begin{aligned}
\int_{\partial\Omega} [\![\kappa\nabla\Phi]\!](\mathbf{x})\Phi(\mathbf{x})\, d\mathbf{x} &= \left(\sigma_s + \sigma_p + \sigma_{\text{ext}}, \Phi\right)_{L^2(\partial\Omega)},\\
2\int_{\partial\Omega} [\![\kappa\nabla\Phi]\!](\mathbf{x})\Phi_{\text{ext}}(\mathbf{x})\, d\mathbf{x} &= 2\left(\sigma_s + \sigma_p + \sigma_{\text{ext}}, \Phi_{\text{ext}}\right)_{L^2(\partial\Omega)},\\
\sum_{j=1}^{N} \int_{\partial\Omega_j} (\kappa_j - \kappa_0)\partial_n\Phi^{jj}(\mathbf{x})\Phi^{jj}(\mathbf{x})\, d\mathbf{x} &= \sum_{j=1}^{N} \left(\sigma_{s,j} + \sigma_{p,j}, \Phi^{jj}\right)_{L^2(\partial\Omega_j)},\\
\int_{\partial\Omega} (\kappa(\mathbf{x}) - \kappa_0)\partial_n\Phi_{\text{ext}}(\mathbf{x})\Phi_{\text{ext}}(\mathbf{x})\, d\mathbf{x} &= -\left(\sigma_{\text{ext}}, \Phi_{\text{ext}}\right)_{L^2(\partial\Omega)},
\end{aligned}
$$

where we remind the reader that $\sigma_{\text{ext}} = -(\kappa - \kappa_0)\partial_n\Phi_{\text{ext}}$. Using the fact that $\lambda, \lambda_{\text{ext}}$ and λ^{jj} are the restrictions on the spheres of the potentials Φ, Φ_{ext}, and Φ^{jj} respectively, we can deduce that

$$
\begin{aligned}
\mathcal{E}^r_{\text{PDE,int}} = &\left(\sigma_s + \sigma_p + \sigma_{\text{ext}},\ \lambda\right)_{L^2(\partial\Omega)} + 2\left(\sigma_s + \sigma_p,\ \lambda_{\text{ext}}\right)_{L^2(\partial\Omega)} + \left(\sigma_{\text{ext}},\ \lambda_{\text{ext}}^{\ell_{\max}}\right)_{L^2(\partial\Omega)} - \sum_{j=1}^{N} \left(\sigma_{s,j} + \sigma_{p,j}, \lambda^{jj}\right)_{L^2(\partial\Omega_j)}\\
&+ \int_{\partial\mathbb{B}_r} \kappa_0 \left(\partial_n\Phi(\mathbf{x})\Phi(\mathbf{x}) - \sum_{j=1}^{N} \partial_n\Phi^{jj}(\mathbf{x})\Phi^{jj}(\mathbf{x})\right) d\mathbf{x} + 2\int_{\partial\mathbb{B}_r} \kappa_0\partial_n\Phi(\mathbf{x})\Phi_{\text{ext}}(\mathbf{x})\, d\mathbf{x} = \mathcal{E}_{\text{int}}.
\end{aligned}
$$

Comparing this final expression with Equation (2) allows us to deduce the required result (28).

■

Next, we will prove Theorem 2.1 which shows that Definition (24) of the approximate electrostatic forces is consistent with the usual notion in the chemistry literature of the forces as the negative sphere-centered gradients of the electrostatic interaction energy. In order to present a concise and well-structured proof, we will first prove two lemmas.

Lemma 2.1 *Let $\lambda_{\ell_{\max}}$ denote the solution to the Galerkin discretisation (18) for a given free charge $\sigma_f = \sigma_s + \sigma_p$ and external electric field $\mathbf{E}_{\text{ext}}$. Additionally, let $\nu_{\ell_{\max}}$ denote the approximate induced surface corresponding to $\lambda_{\ell_{\max}}$ and let $\mathbf{E}^i_{\text{exc}}$, $i \in \{1,\dots,N\}$ denote the i-excluded electric fields generated by $\nu_{\ell_{\max}}$ as defined through Definition (17). Then for each $i \in \{1,\dots,N\}$ it holds that*

$$\frac{1}{2}\nabla_{\mathbf{x}_i}\Big(\sigma_s+\sigma_p+\sigma_{\text{ext}},\lambda_{\ell_{\max}}\Big)_{L^2(\partial\Omega)} = -\kappa_0\big(\nu_{\ell_{\max}},\mathbf{E}^i_{\text{exc}}\big)_{L^2(\partial\Omega_i)} + \big(\nabla_{\mathbf{x}_i}\sigma_{\text{ext}},\lambda_{\ell_{\max}}\big)_{L^2(\partial\Omega_i)}.$$

Proof: Let $i \in \{1,\dots,N\}$ be fixed. A simple application of the product rule yields that

$$\begin{aligned}\frac{1}{2}\nabla_{\mathbf{x}_i}\Big(\sigma_s+\sigma_p+\sigma_{\text{ext}},\lambda_{\ell_{\max}}\Big)_{L^2(\partial\Omega)} &= \frac{1}{2}\Big(\nabla_{\mathbf{x}_i}\left(\sigma_s+\sigma_p+\sigma_{\text{ext}}\right),\lambda_{\ell_{\max}}\Big)_{L^2(\partial\Omega)}\\ &+\frac{1}{2}\Big(\sigma_s+\sigma_p+\sigma_{\text{ext}},\nabla_{\mathbf{x}_i}\lambda_{\ell_{\max}}\Big)_{L^2(\partial\Omega)}.\end{aligned}$$

Using the fact that both σ_s and σ_p are independent of changes in the locations $\{\mathbf{x}_i\}_{i=1}^N$ of the sphere centres locations,[2] we further obtain that

$$\frac{1}{2}\nabla_{\mathbf{x}_i}\Big(\sigma_s+\sigma_p+\sigma_{\text{ext}},\lambda_{\ell_{\max}}\Big)_{L^2(\partial\Omega)} = \frac{1}{2}\Big(\nabla_{\mathbf{x}_i}\sigma_{\text{ext}},\lambda_{\ell_{\max}}\Big)_{L^2(\partial\Omega)} + \frac{1}{2}\Big(\sigma_s+\sigma_p+\sigma_{\text{ext}},\nabla_{\mathbf{x}_i}\lambda_{\ell_{\max}}\Big)_{L^2(\partial\Omega)}.$$

Finally, it is straightforward to see that in fact

$$\frac{1}{2}\Big(\nabla_{\mathbf{x}_i}\sigma_{\text{ext}},\lambda_{\ell_{\max}}\Big)_{L^2(\partial\Omega)} = \frac{1}{2}\Big(\nabla_{\mathbf{x}_i}\sigma_{\text{ext}},\lambda_{\ell_{\max}}\Big)_{L^2(\partial\Omega_i)}$$

so that we obtain the expression

$$\frac{1}{2}\nabla_{\mathbf{x}_i}\Big(\big(\sigma_s+\sigma_p+\sigma_{\rm ext}\big),\lambda_{\ell_{\max}}\Big)_{L^2(\partial\Omega)}=\frac{1}{2}\Big(\nabla_{\mathbf{x}_i}\sigma_{\rm ext},\lambda_{\ell_{\max}}\Big)_{L^2(\partial\Omega_i)}+\frac{1}{2}\Big(\big(\sigma_s+\sigma_p+\sigma_{\rm ext}\big),\nabla_{\mathbf{x}_i}\lambda_{\ell_{\max}}\Big)_{L^2(\partial\Omega)}. \tag{3}$$

Consequently, it remains to compute the sphere-centred gradient of $\lambda_{\ell_{\max}}$. This is a slightly technical task so to aid the subsequent exposition, we first introduce some additional notation.

Notation: We define the vectors and matrices $\boldsymbol{\sigma},\boldsymbol{\sigma}^{\rm ext},\boldsymbol{DtN}^{\kappa}$ and $\boldsymbol{V}$ as

$$\begin{aligned}
[\boldsymbol{\sigma}_i]_\ell^m &:= \Big(\sigma_s+\sigma_p,\mathcal{Y}_{\ell m}^i\Big)_{L^2(\partial\Omega_i)},\\
[\boldsymbol{\sigma}_i^{\rm ext}]_\ell^m &:= \Big(\sigma_{\rm ext},\mathcal{Y}_{\ell m}^i\Big)_{L^2(\partial\Omega_i)},\\
[\boldsymbol{DtN}_{ij}^{\kappa}]_{\ell\ell'}^{mm'} &:= \delta_{ij}\left(\frac{\kappa_j-\kappa_0}{\kappa_0}\mathrm{DtN}\mathcal{Y}_{\ell'm'}^j,\mathcal{Y}_{\ell m}^i\right)_{L^2(\partial\Omega_i)},\\
[\boldsymbol{V}_{ij}]_{\ell\ell'}^{mm'} &:= \Big(\mathcal{V}\mathcal{Y}_{\ell'm'}^j,\mathcal{Y}_{\ell m}^i\Big)_{L^2(\partial\Omega_i)},
\end{aligned}$$

where $i,j\in\{1,\dots,N\}$, $\ell,\ell'\in\{0,\dots,\ell_{\max}\}$ and $|m|\le\ell$, $|m'|\le\ell'$. Additionally, we recall that the Galerkin discretisation (18) is equivalent to the linear system of equations

$$\boldsymbol{A}\boldsymbol{\lambda}:=(\boldsymbol{I}-\boldsymbol{V}\,\boldsymbol{DtN}^{\kappa})\boldsymbol{\lambda}=\boldsymbol{F}, \tag{4}$$

where $\boldsymbol{\lambda}$ and $\boldsymbol{F}$ are defined by (20) and (21) respectively.

Equipped with the notation introduced above, we now take the gradient on both sides of Equation (4). Using the product rule together with the fact that the Dirichlet-to-Neumann

map is independent of changes in the locations $\{\mathbf{x}_i\}_{i=1}^N$ of the sphere centers, we obtain that

$$\nabla_{\mathbf{x}_i}\boldsymbol{\lambda} + \left(\nabla_{\mathbf{x}_i}\boldsymbol{V}\right)\boldsymbol{DtN}^{\kappa}\boldsymbol{\lambda} + \boldsymbol{V}\boldsymbol{DtN}^{\kappa}\nabla_{\mathbf{x}_i}\boldsymbol{\lambda} = \frac{1}{\kappa_0}\left(\nabla_{\mathbf{x}_i}\boldsymbol{V}\right)\left(\boldsymbol{\sigma} + \boldsymbol{\sigma}^{\text{ext}}\right) + \frac{1}{\kappa_0}\boldsymbol{V}\nabla_{\mathbf{x}_i}\boldsymbol{\sigma}^{\text{ext}},$$

or equivalently, after collecting terms

$$\boldsymbol{A}\nabla_{\mathbf{x}_i}\boldsymbol{\lambda} = \frac{1}{\kappa_0}\left(\nabla_{\mathbf{x}_i}\boldsymbol{V}\right)\left(\boldsymbol{\sigma} + \boldsymbol{\sigma}^{\text{ext}} - \kappa_0\boldsymbol{DtN}^{\kappa}\boldsymbol{\lambda}\right) + \frac{1}{\kappa_0}\boldsymbol{V}\nabla_{\mathbf{x}_i}\boldsymbol{\sigma}^{\text{ext}}. \tag{5}$$

Next, recalling that $\nu_{\ell_{\max}}$ satisfies Equation (23), it is easy to deduce that

$$\frac{1}{\kappa_0}\left(\nabla_{\mathbf{x}_i}\boldsymbol{V}\right)\left(\boldsymbol{\sigma} + \boldsymbol{\sigma}^{\text{ext}} - \kappa_0\boldsymbol{DtN}^{\kappa}\boldsymbol{\lambda}\right) = \left(\nabla_{\mathbf{x}_i}\boldsymbol{V}\right)\boldsymbol{\nu},$$

where

$$[\boldsymbol{\nu}_i]_{\ell}^m := \left(\nu_{\ell_{\max}}, \mathcal{Y}_{\ell m}^i\right)_{L^2(\partial\Omega_i)},$$

with indices $i \in \{1,\dots,N\}$, $\ell \in \{0,\dots,\ell_{\max}\}$ and $|m| \le \ell$. We therefore conclude from Equation (5) that

$$\nabla_{\mathbf{x}_i}\boldsymbol{\lambda} = \boldsymbol{A}^{-1}\left(\left(\nabla_{\mathbf{x}_i}\boldsymbol{V}\right)\boldsymbol{\nu}\right) + \frac{1}{\kappa_0}\boldsymbol{A}^{-1}\left(\boldsymbol{V}\nabla_{\mathbf{x}_i}\boldsymbol{\sigma}^{\text{ext}}\right).$$

Recalling now the last term on the right-hand side of Equation (3), we deduce that

$$\begin{aligned}
\frac{1}{2}\left(\sigma_s + \sigma_p + \sigma_{\text{ext}}, \nabla_{\mathbf{x}_i}\lambda_{\ell_{\max}}\right)_{L^2(\partial\Omega)} &= \frac{1}{2}\left(\boldsymbol{\sigma} + \boldsymbol{\sigma}^{\text{ext}}, \nabla_{\mathbf{x}_i}\boldsymbol{\lambda}\right)_{\ell^2} \\
&= \frac{1}{2}\left(\boldsymbol{\sigma} + \boldsymbol{\sigma}^{\text{ext}}, \boldsymbol{A}^{-1}\left(\left(\nabla_{\mathbf{x}_i}\boldsymbol{V}\right)\boldsymbol{\nu}\right)\right)_{\ell^2} + \frac{1}{2}\left(\boldsymbol{\sigma} + \boldsymbol{\sigma}^{\text{ext}}, \frac{1}{\kappa_0}\boldsymbol{A}^{-1}\left(\boldsymbol{V}\nabla_{\mathbf{x}_i}\boldsymbol{\sigma}^{\text{ext}}\right)\right)_{\ell^2} \\
&= \frac{1}{2}\left(\left(\boldsymbol{A}^{\boldsymbol{T}}\right)^{-1}\left(\boldsymbol{\sigma} + \boldsymbol{\sigma}^{\text{ext}}\right), \left(\nabla_{\mathbf{x}_i}\boldsymbol{V}\right)\boldsymbol{\nu}\right)_{\ell^2} + \frac{1}{2}\frac{1}{\kappa_0}\left(\left(\boldsymbol{A}^{\boldsymbol{T}}\right)^{-1}\left(\boldsymbol{\sigma} + \boldsymbol{\sigma}^{\text{ext}}\right), \boldsymbol{V}\nabla_{\mathbf{x}_i}\boldsymbol{\sigma}^{\text{ext}}\right)_{\ell^2}.
\end{aligned}$$

Next, a direct calculation and comparison with the Galerkin discretisation (18) reveals that

$$\left(\boldsymbol{A}^{\boldsymbol{T}}\right)^{-1}\left(\boldsymbol{\sigma}+\boldsymbol{\sigma}^{\text{ext}}\right)=\kappa_0\boldsymbol{\nu}.$$

Using the definition of $\nu_{\ell_{\max}}$ as given by Equation (22), we obtain that

$$\begin{aligned}\frac{1}{2}\left(\sigma_s+\sigma_p+\sigma_{\text{ext}},\nabla_{\mathbf{x}_i}\lambda_{\ell_{\max}}\right)_{L^2(\partial\Omega)} &= \frac{1}{2}\kappa_0\left(\boldsymbol{\nu},\left(\nabla_{\mathbf{x}_i}\boldsymbol{V}\right)\boldsymbol{\nu}\right)_{\ell^2}+\frac{1}{2}\left(\boldsymbol{\nu},\boldsymbol{V}\nabla_{\mathbf{x}_i}\boldsymbol{\sigma}^{\text{ext}}\right)_{\ell^2}\\ &= \frac{1}{2}\kappa_0\left(\boldsymbol{\nu},\left(\nabla_{\mathbf{x}_i}\boldsymbol{V}\right)\boldsymbol{\nu}\right)_{\ell^2}+\frac{1}{2}\left(\boldsymbol{\lambda},\nabla_{\mathbf{x}_i}\boldsymbol{\sigma}^{\text{ext}}\right)_{\ell^2}\\ &= \frac{1}{2}\kappa_0\left(\nu_{\ell_{\max}},\left(\nabla_{\mathbf{x}_i}\mathcal{V}\right)\nu_{\ell_{\max}}\right)_{L^2(\partial\Omega)}+\frac{1}{2}\left(\lambda_{\ell_{\max}},\nabla_{\mathbf{x}_i}\sigma_{\text{ext}}\right)_{L^2(\partial\Omega)}\\ &= \frac{1}{2}\kappa_0\left(\nu_{\ell_{\max}},\left(\nabla_{\mathbf{x}_i}\mathcal{V}\right)\nu_{\ell_{\max}}\right)_{L^2(\partial\Omega)}+\frac{1}{2}\left(\lambda_{\ell_{\max}},\nabla_{\mathbf{x}_i}\sigma_{\text{ext}}\right)_{L^2(\partial\Omega_i)} \quad (6)\end{aligned}$$

Finally, a direct but tedious computation can be used to show that[2,3]

$$\frac{1}{2}\kappa_0\left(\nu_{\ell_{\max}},\left(\nabla_{\mathbf{x}_i}\mathcal{V}\right)\nu_{\ell_{\max}}\right)_{L^2(\partial\Omega)}=-\kappa_0\left(\nu_{\ell_{\max}},\mathbf{E}^i_{\text{exc}}\right)_{L^2(\partial\Omega_i)}. \quad (7)$$

Combining therefore the developments (6) and (7) with Equation (3) now completes the proof. ■

Lemma 2.2 *For a given external electric field* $\mathbf{E}_{\text{ext}}=-\nabla\Phi_{\text{ext}}\in L^2_{\text{loc}}(\mathbb{R}^3)$*, let* $\sigma_{\text{ext}}=-(\kappa-\kappa_0)\partial_n\Phi_{\text{ext}}$*, and let* $\psi\in H^{\frac{1}{2}}(\partial\Omega)$ *be arbitrary. Then for each* $i\in\{1,\ldots,N\}$ *it holds that*

$$\left(\nabla_{\mathbf{x}_i}\sigma_{\text{ext}},\Psi\right)_{L^2(\partial\Omega_i)}=-(\kappa_i-\kappa_0)\left(\nabla_{\mathbf{x}_i}\lambda_{\text{ext}},\text{DtN}\Psi\right)_{L^2(\partial\Omega_i)}. \quad (8)$$

Proof: Recall the notation $\lambda_{\text{ext}}:=\Phi_{\text{ext}}|_{\partial\Omega}\in H^{\frac{1}{2}}(\partial\Omega)$ and let $[\lambda_{\text{ext,i}}]^m_\ell$ and $[\Psi_i]^m_\ell$, $\ell\in\mathbb{N}_0$, $|m|\leq\ell$ denote the local spherical harmonics expansion coefficients of λ_{ext} and Ψ on the sphere $\partial\Omega_i$.

Since Φ_{ext} is harmonic in $\mathbb{R}^3$ and therefore in particular on $\overline{\Omega_i}$, it follows that we can write

$$\begin{aligned}\left(\nabla_{\mathbf{x}_i}\sigma_{\text{ext}},\Psi\right)_{L^2(\partial\Omega_i)} &= -(\kappa_i-\kappa_0)\left(\nabla_{\mathbf{x}_i}\partial_n\Phi_{\text{ext}},\Psi\right)_{L^2(\partial\Omega_i)}\\ &= -(\kappa_i-\kappa_0)\left(\text{DtN}\lambda_{\text{ext}},\Psi\right)_{L^2(\partial\Omega_i)}\\ &= -(\kappa_i-\kappa_0)r_i^2\sum_{\ell=0}^{\ell=\infty}\sum_{m=-\ell}^{m=+\ell}\left(\nabla_{\mathbf{x}_i}\frac{\ell}{r_i}[\lambda_{\text{ext,i}}]_\ell^m\right)[\Psi_i]_\ell^m\\ &= -(\kappa_i-\kappa_0)r_i^2\sum_{\ell=0}^{\ell=\infty}\sum_{m=-\ell}^{m=+\ell}\left(\nabla_{\mathbf{x}_i}[\lambda_{\text{ext,i}}]_\ell^m\right)\left(\frac{\ell}{r_i}[\Psi_i]_\ell^m\right)\\ &= -(\kappa_i-\kappa_0)\left(\nabla_{\mathbf{x}_i}\lambda_{\text{ext}},\text{DtN}\Psi\right)_{L^2(\partial\Omega_i)}.\end{aligned}$$

■

2.2 Proof of Theorem 2.1

We are now ready to state the proof of Theorem 2.1. Before proceeding to the proof, let us simply remark that the relation (26) in Theorem 2.1 remains true if exact quantities are considered, i.e., if the force defined by (24) is built upon the exact induced charge ν being solution to the BIE (7) and where the energy corresponds to $\mathcal{E}_{\text{int}}$ as defined by (27).

Let $i \in \{1,\dots,N\}$ be fixed. By the definition of the approximate electrostatic interaction energy, we have

$$\begin{aligned}-\nabla_{\mathbf{x}_i}\mathcal{E}_{\text{int}}^{\ell_{\max}} = &\underbrace{-\frac{1}{2}\nabla_{\mathbf{x}_i}\left(\sigma_s+\sigma_p+\sigma_{\text{ext}},\ \lambda_{\ell_{\max}}\right)_{L^2(\partial\Omega)}}_{:=(\text{I})}\underbrace{-\nabla_{\mathbf{x}_i}\left(\sigma_s+\sigma_p,\ \lambda_{\text{ext}}^{\ell_{\max}}\right)_{L^2(\partial\Omega)}}_{:=(\text{II})}\\ &\underbrace{-\frac{1}{2}\nabla_{\mathbf{x}_i}\left(\sigma_{\text{ext}},\ \lambda_{\text{ext}}^{\ell_{\max}}\right)_{L^2(\partial\Omega)}}_{:=(\text{III})}+\underbrace{\frac{1}{2}\nabla_{\mathbf{x}_i}\sum_{j=1}^{N}\left(\sigma_{s,j}+\sigma_{p,j},\lambda_{\ell_{\max}}^{jj}\right)_{L^2(\partial\Omega_j)}}_{:=(\text{IV})}.\end{aligned}$$

We now simplify each of the terms (I), (II), (III), and (IV). First, we observe that the self energy term (IV) is defined entirely through functions that are independent of changes in the

location of the center $\mathbf{x}_i$ of the sphere $\partial\Omega_i$, even in the case $j = i$. This can be seen by noticing that $\left(\sigma_{s,i} + \sigma_{p,i}, \lambda^{ii}_{\ell_{\max}}\right)_{L^2(\partial\Omega_i)}$ remains constant as one displaces $\mathbf{x}_i$ by any translation. Consequently, we obtain that (IV) $\equiv 0$.

The term (I) can be simplified using Lemmas 2.1 and 2.2 as

$$\begin{aligned}
\text{(I)} &= -\frac{1}{2}\nabla_{\mathbf{x}_i}\left(\sigma_s + \sigma_p + \sigma_{\text{ext}}, \lambda_{\ell_{\max}}\right)_{L^2(\partial\Omega)} \\
&= \kappa_0\left(\nu_{\ell_{\max}}, \mathbf{E}^i_{\text{exc}}\right)_{L^2(\partial\Omega_i)} - \left(\nabla_{\mathbf{x}_i}\sigma_{\text{ext}}, \lambda_{\ell_{\max}}\right)_{L^2(\partial\Omega_i)} && \text{(Using Lemma 2.1)} \\
&= \underbrace{\kappa_0\left(\nu_{\ell_{\max}}, \mathbf{E}^i_{\text{exc}}\right)_{L^2(\partial\Omega_i)}}_{:=\text{(IA)}} + \underbrace{(\kappa_i - \kappa_0)\left(\nabla_{\mathbf{x}_i}\lambda_{\text{ext}}, \text{DtN}\lambda_{\ell_{\max}}\right)_{L^2(\partial\Omega_i)}}_{:=\text{(IB)}}. && \text{(Using Lemma 2.2)} \quad (9)
\end{aligned}$$

Next, we simplify the term (IB). Indeed, a direct calculation shows that

$$\begin{aligned}
\text{(IB)} &= (\kappa_i - \kappa_0)\left(\nabla_{\mathbf{x}_i}\lambda_{\text{ext}}, \text{DtN}\lambda_{\ell_{\max}}\right)_{L^2(\partial\Omega_i)} = (\kappa_i - \kappa_0)\int_{\partial\Omega_i}(\nabla_{\mathbf{x}_i}\Phi_{\text{ext}})\text{DtN}\lambda_{\ell_{\max}}\, d\mathbf{x} \\
&= (\kappa_i - \kappa_0)\int_{\partial\Omega_i}(\nabla_{\mathbf{x}}\Phi_{\text{ext}})\text{DtN}\lambda_{\ell_{\max}}\, d\mathbf{x} = -(\kappa_i - \kappa_0)\int_{\partial\Omega_i}\mathbf{E}_{\text{ext}}\text{DtN}\lambda_{\ell_{\max}}\, d\mathbf{x} \\
&= -(\kappa_i - \kappa_0)\left(\text{DtN}\lambda_{\ell_{\max}}, \mathbf{E}_{\text{ext}}\right)_{L^2(\partial\Omega_i)}, \qquad (10)
\end{aligned}$$

where the second line follows from a similar calculation as done to obtain Equation (7).

In order to simplify the term (II), we again recall that the free charges σ_s, σ_p are independent of changes in the location of the center $\mathbf{x}_i$ of the sphere $\partial\Omega_i$. Consequently, we obtain

$$\text{(II)} = -\nabla_{\mathbf{x}_i}\left(\sigma_s + \sigma_p, \lambda^{\ell_{\max}}_{\text{ext}}\right)_{L^2(\partial\Omega)} = -\left(\sigma_s + \sigma_p,\ \nabla_{\mathbf{x}_i}\lambda^{\ell_{\max}}_{\text{ext}}\right)_{L^2(\partial\Omega)} = -\left(\sigma_s + \sigma_p,\ \nabla_{\mathbf{x}_i}\lambda^{\ell_{\max}}_{\text{ext}}\right)_{L^2(\partial\Omega)_i}.$$

Therefore, using a calculation similar to the one used to obtain Equation (10), we deduce

that

$$(\text{II}) = -\big(\sigma_s + \sigma_p,\, \nabla_{\mathbf{x}_i} \lambda_{\text{ext}}^{\ell_{\max}}\big)_{L^2(\partial\Omega_i)} = \big(\sigma_s^{\ell_{\max}} + \sigma_p^{\ell_{\max}},\, \mathbf{E}_{\text{ext}}\big)_{L^2(\partial\Omega_i)}, \tag{11}$$

where $\sigma_s^{\ell_{\max}}$ and $\sigma_p^{\ell_{\max}}$ are the best approximations in $W^{\ell_{\max}}$ of σ_s and σ_p respectively.

Next, we attempt to simplify the term (III). A simple application of the product rule together with Lemma 2.2 yields that

$$\begin{aligned}
(\text{III}) =& -\frac{1}{2}\nabla_{\mathbf{x}_i}\big(\sigma_{\text{ext}},\, \lambda_{\text{ext}}^{\ell_{\max}}\big)_{L^2(\partial\Omega)} = -\frac{1}{2}\nabla_{\mathbf{x}_i}\big(\sigma_{\text{ext}},\, \lambda_{\text{ext}}^{\ell_{\max}}\big)_{L^2(\partial\Omega_i)}\\
=& -\frac{1}{2}\big(\nabla_{\mathbf{x}_i}\sigma_{\text{ext}},\, \lambda_{\text{ext}}^{\ell_{\max}}\big)_{L^2(\partial\Omega_i)} - \frac{1}{2}\big(\sigma_{\text{ext}},\, \nabla_{\mathbf{x}_i}\lambda_{\text{ext}}^{\ell_{\max}}\big)_{L^2(\partial\Omega_i)}\\
=&(\kappa_i - \kappa_0)\big(\text{DtN}\lambda_{\text{ext}}, \nabla_{\mathbf{x}_i}\lambda_{\text{ext}}^{\ell_{\max}}\big)_{L^2(\partial\Omega_i)} - \frac{1}{2}\big(\sigma_{\text{ext}},\, \nabla_{\mathbf{x}_i}\lambda_{\text{ext}}^{\ell_{\max}}\big)_{L^2(\partial\Omega_i)}\\
=& -\big(\sigma_{\text{ext}},\, \nabla_{\mathbf{x}_i}\lambda_{\text{ext}}^{\ell_{\max}}\big)_{L^2(\partial\Omega_i)}.
\end{aligned}$$

Once again, a direct calculation of the form used to obtain Equation (10) allows us to conclude that

$$(\text{III}) = -\big(\sigma_{\text{ext}},\, \nabla_{\mathbf{x}_i} \lambda_{\text{ext}}^{\ell_{\max}}\big)_{L^2(\partial\Omega_i)} = \big(\sigma_{\text{ext}}^{\ell_{\max}},\, \mathbf{E}_{\text{ext}}\big)_{L^2(\partial\Omega_i)}. \tag{12}$$

Combining now Equations (9), (10), (11), and (12) we obtain that

$$
\begin{aligned}
-\nabla_{\mathbf{x}_i}\mathscr{E}_{\text{int}}^{\ell_{\max}} &= \text{(IA)} + \text{(IB)} + \text{(II)} + \text{(III)} \\
&= \kappa_0\big(\nu_{\ell_{\max}}, \mathbf{E}_{\text{exc}}^i\big)_{L^2(\partial\Omega_i)} - (\kappa_i - \kappa_0)\big(\text{DtN}\lambda_{\ell_{\max}}, \mathbf{E}_{\text{ext}}\big)_{L^2(\partial\Omega_i)} \\
&+ \big(\sigma_s^{\ell_{\max}} + \sigma_p^{\ell_{\max}},\ \mathbf{E}_{\text{ext}}\big)_{L^2(\partial\Omega_i)} + \big(\sigma_{\text{ext}}^{\ell_{\max}},\ \mathbf{E}_{\text{ext}}\big)_{L^2(\partial\Omega_i)} \\
&= \kappa_0\big(\nu_{\ell_{\max}}, \mathbf{E}_{\text{exc}}^i\big)_{L^2(\partial\Omega_i)} + \big(-(\kappa_i - \kappa_0)\text{DtN}\lambda_{\ell_{\max}} + \sigma_s^{\ell_{\max}} + \sigma_p^{\ell_{\max}} + \sigma_{\text{ext}}^{\ell_{\max}}, \mathbf{E}_{\text{ext}}\big)_{L^2(\partial\Omega_i)} \\
&= \kappa_0\big(\nu_{\ell_{\max}}, \mathbf{E}_{\text{exc}}^i\big)_{L^2(\partial\Omega_i)} + \kappa_0\big(\nu_{\ell_{\max}}, \mathbf{E}_{\text{ext}}\big)_{L^2(\partial\Omega_i)}.
\end{aligned}
$$

where the last equality follows from Equation (23). This completes the proof. ■

References

(1) Sauter, S.; Schwab, C. *Boundary Element Methods*; Springer-Verlag, Berlin, 2010.

(2) Hassan, M. Mathematical Analysis of Boundary Integral Equations and Domain Decomposition Methods with Applications in Polarisable Electrostatics. Ph.D. thesis, RWTH Aachen University, 2020.

(3) Hassan, M.; Stamm, B. A Linear Scaling in Accuracy Numerical Method for Computing the Electrostatic Forces in the N-Body Dielectric Spheres Problem. *Commun. Comput. Phys.* **2021**, *29*, 319–356.
